# mHealth-Supported Exercise Rehabilitation to Reverse Frailty After Autologous Transplantation in Multiple Myeloma: Randomized Controlled Trial

**DOI:** 10.2196/87628

**Published:** 2026-05-21

**Authors:** Kyuwan Lee, Justin Shamunee, Haehyun Lee, Xinyi Du, Lanie Lindenfeld, Amrita Krishnan, Nitya Nathwani, F Lennie Wong, Saro Armenian

**Affiliations:** 1 Kinesiology & Sports Studies Ewha Womans University Seoul, Seoul Republic of Korea; 2 Smidt Heart Institute Cedars-Sinai Medical Center Los Angeles, CA United States; 3 University of Nevada, Las Vegas Las Vegas, NV United States; 4 Population Sciences City of Hope Duarte, CA United States

**Keywords:** mobile health exercise, mHealth exercise, multiple myeloma, frailty, autologous hematopoietic cell transplantation

## Abstract

**Background:**

Frailty is highly prevalent in survivors of multiple myeloma (MM) after autologous hematopoietic cell transplantation and is associated with poor functional recovery and adverse clinical outcomes. Although exercise is known to improve physical function, traditional center-based rehabilitation models are often inaccessible to this population during early posttransplant recovery. Mobile health (mHealth)–supported exercise may offer a scalable alternative; however, evidence in hematologic malignancies remains limited.

**Objective:**

This study aimed to evaluate the effects of a 16-week mHealth-supported exercise rehabilitation program on frailty phenotype and physical function in survivors of MM within 180 days after autologous hematopoietic cell transplantation.

**Methods:**

In this single-center randomized controlled trial, participants who self-reported as prefrail or frail were randomized 1:1 to an mHealth-supported exercise group (n=16) or usual care control (n=16). Remote assessments were conducted at baseline (week 0), midpoint (week 9), and follow-up (week 17). The intervention consisted of 8 weeks of supervised tele-exercise (3 sessions/week, 50 minutes/session), followed by 8 weeks of independent home-based exercise using the same mHealth platform. Exercise intensity was prescribed using a repetitions-in-reserve–based rating of perceived exertion approach with symptom-guided progression. The primary outcome was change in the 5-component Fried frailty phenotype score (0-5). Secondary outcomes included Short Physical Performance Battery components, chair stand time, gait speed, and handgrip strength. Intention-to-treat analyses were conducted using generalized estimating equations to evaluate between-group differences over time.

**Results:**

Participants had a mean age of 64.6 (SD 7.1) years and were enrolled a mean of 136 (SD 36.3) days posttransplant. At baseline, 94% (30/32) of participants were classified as frail. Adherence to the supervised sessions was 85% (326/384 sessions), and adherence during the unsupervised phase was 78% (298/384 sessions). The exercise group demonstrated a significantly greater reduction in frailty score compared with control from baseline to week 17 (*P*<.001). Between-group difference estimates showed a clinically meaningful improvement favoring exercise at both week 9 and week 17 (*P*<.001). Chair stand time improved significantly in the exercise group compared with control, with faster completion times observed at week 9 and sustained through week 17 (*P*=.002). Improvements in other Short Physical Performance Battery components and handgrip strength favored the exercise group but did not reach statistical significance. No serious adverse events occurred.

**Conclusions:**

A 16-week mHealth-supported, progressively prescribed exercise rehabilitation program was feasible, safe, and effective in reversing frailty phenotype and improving functional mobility in survivors of MM early after autologous transplantation. This approach provides a scalable model for delivering structured rehabilitation during a high-risk recovery window. Larger trials incorporating attention-matched controls and longer follow-up are warranted.

**Trial Registration:**

ClinicalTrials.gov NCT05142371; https://clinicaltrials.gov/study/NCT05142371

## Introduction

### Background and Rationale

Autologous hematopoietic cell transplantation (HCT) improves progression-free survival in patients with multiple myeloma (MM) [1]. Advances in treatment approaches, coupled with improved access to HCT, have contributed to a growing number of patients with MM undergoing HCT annually. In fact, MM is the most common indication for HCT, with >7000 patients undergoing autologous HCT annually in the United States alone [2]. However, despite improvements in disease-related outcomes, patients with MM who undergo HCT remain at high risk of experiencing frailty after HCT, attributed to pre-HCT treatment exposures, HCT-related conditioning therapies, and post-HCT acute toxicities [3,4].

The frailty phenotype is characterized by the presence of 3 or more of the following characteristics: underweight, exhaustion, low energy expenditure, slow gait speed, and weak muscle strength [3,5,6]. Two-thirds of patients with MM are estimated to be frail at diagnosis or shortly thereafter, and the prevalence of frailty increases after HCT [7]. In an International Myeloma Working Group report, frailty was associated with significantly worse overall survival and a higher rate of treatment discontinuation (31.2% vs 16.5%) [8]. This underscores the need to develop innovative strategies to mitigate frailty in patients with MM and its associated morbidity and mortality following HCT.

Supervised exercise-based interventions have been shown to improve physical function and frailty in patients with chronic health conditions, including cardiovascular diseases and cancer [9,10]. Traditionally, supervised exercise interventions have been delivered in person at specialized centers, an approach that poses logistical challenges and limits access for many patients. Supervised prescriptive mobile health (mHealth) exercise interventions have emerged as alternatives to in-person approaches, offering the convenience of anytime and anywhere access to patients, regardless of geographic boundaries [11]. Studies in patients with other cancer types, such as the breast and endometrium, have shown that an mHealth exercise is effective in improving physical function, BMI, and health-related quality of life [12,13]. There is a paucity of information on the safety and efficacy of mHealth exercise intervention in prefrail and frail patients with MM.

### Objective and Hypothesis

To address this limitation, we conducted a randomized controlled trial evaluating the effectiveness of an mHealth exercise intervention aimed at improving frailty and physical function in patients with MM shortly after undergoing HCT. We hypothesized that a 16-week mHealth exercise intervention would significantly improve frailty and physical function in these patients.

## Methods

### Study Design and Setting

This was a single-center randomized control trial conducted at City of Hope (COH). The trial was registered at ClinicalTrials.gov (NCT05142371) on October 29, 2021. We conducted a 16-week mHealth intervention that included baseline (week 0) and postintervention (week 9 and week 17) assessments of physical function and frailty phenotype. All study procedures, including screening, enrollment, intervention, and data collection, were performed using an mHealth-based approach, eliminating the need for participants to be assessed at a center. This study adheres to the CONSORT (Consolidated Standards of Reporting Trials) guidelines for reporting randomized controlled trials.

### Participants and Eligibility Criteria

The participant eligibility criteria are shown in Textbox 1.

**Participant eligibility criteria.**

**Inclusion criteria**

- Diagnosis of multiple myeloma
- Aged >18 years at enrollment
- Prefrail or frail status (ie, Fried criteria: clinically underweight [BMI <18.5 kg/m^2^], exhaustion, low energy expenditure, slow walking speed, and muscle weakness), with the presence of 2 of 5 indexes classified as prefrail and ≥3 of 5 indexes classified as frail [14]
- <180 days after hematopoietic cell transplantation
- Physically able and willing to complete all study procedures
- English speaking

**Exclusion criteria**

- Had clinically significant or active cardiovascular disease (eg, unstable angina and uncontrolled arrhythmia)
- Had contraindications to exercise (acute infectious disease or unstable bone lesions)
- Were recovering from a recent injury or were physically injured in the past 6 months, whereby participation in rigorous exercise may not be appropriate
- Were already participating in regular, structured exercise (>60 minutes/week)

### Screening, Recruitment, and Randomization

Prescreening of potentially eligible patients was performed using an established institutional review board–approved COH protocol for long-term follow-up after HCT [15]. During telephone prescreening, participants were asked simple mobility and fatigue screening questions, including whether they were able to ambulate independently without assistance and whether they experienced persistent fatigue or low physical activity. Frailty status was formally confirmed during the baseline remote assessment prior to randomization. Individuals self-reporting characteristics consistent with prefrailty or frailty were invited for formal remote assessment. Once potentially eligible patients were identified, we notified their primary hematologist to obtain clearance prior to our outreach. Potentially eligible patients were then contacted by phone to assess their willingness to participate and to screen for patient-reported symptoms related to prefrailty or frailty. If potentially eligible patients expressed interest in the study, a trained clinical research assistant conducted detailed discussions with the patients over the phone regarding the additional study inclusion and exclusion criteria. Once patients were found to be eligible for participation, they provided informed consent using a web-based consent form (DocuSign Inc). Participants were randomly assigned in a 1:1 ratio to either the mHealth exercise group or the control group using a computer-generated random allocation sequence. The sequence was prepared by an independent statistician not involved in study conduct. Allocation concealment was maintained, as participants were enrolled by the study coordinator, while group assignments were implemented by a separate investigator who was unaware of the allocation sequence.

### Intervention: mHealth-Supported Exercise Intervention

#### Intensity Prescription and Progression

Exercise intensity during resistance training was prescribed using a repetitions-in-reserve–based rating of perceived exertion (RPE) scale [16]. Participants were instructed to perform exercises at an intensity corresponding to RPE 7 to 8, indicating that they could complete approximately 2 to 3 additional repetitions before fatigue. If participants reported that they could perform >4 additional repetitions (RPE ≤6), resistance or repetitions were increased. Conversely, if participants could perform fewer than 1 to 2 additional repetitions (RPE ≥9) or reported excessive fatigue, discomfort, or dizziness, the exercise was modified by reducing resistance, repetitions, or range of motion and increasing rest intervals. Progression followed a standardized symptom-guided approach. When participants completed all prescribed sets at RPE ≤7 for 2 consecutive sessions, exercise difficulty was progressed by increasing repetitions, adding sets, or advancing resistance band tension and balance challenge. This allowed individualized progression while maintaining safety in the early posttransplant period. Details of the intervention strategies used have been previously published [17]. Exercise equipment, including resistance bands, resistance loop bands, and exercise mats, was shipped directly to participants from COH. The exercise intervention consisted of 2 phases. The first phase offered each participant 24 supervised exercise sessions, each lasting 50 minutes and held 3 times per week for 8 weeks. All sessions in the first 8 weeks were supervised remotely via videoconferencing. Participants could reschedule or make up sessions if they were unable to attend as planned. Their adherence to the program was tracked on the platform. After completing the initial 8-week intervention, the second phase encouraged participants to continue exercising independently without direct monitoring by the exercise trainer for the subsequent 8 weeks until the follow-up physical assessment (week 17). Participants in the control group were asked to maintain their current lifestyle for 17 weeks. They were later offered the same mHealth exercise intervention as a courtesy, though it was not mandatory for completing the study procedures.

#### Sample Exercise Plan

A representative example of a supervised exercise session structure, including balance, strength, and core components with repetitions-in-reserve–based RPE targets and progression rules, is provided in Multimedia Appendix 1 to facilitate reproducibility of the intervention.

#### Outcomes and Remote Assessments

We used a commercially available mHealth exercise platform (Moterum Technologies) that offers exercise videos, physical activity monitoring, and automatic data collection from wearable devices. We sent gait sensors (for the hip, right foot, and left foot), a hand dynamometer, measuring tape, and blue tape to each participant’s home via mail. This equipment was used to assist in measuring physical function and frailty phenotype outcomes before and after the intervention in both groups.

#### Frailty Phenotype (Primary Outcome): Components and Cutoff Values

Frailty was assessed using the 5-component Fried phenotype (score range 0-5), with 1 point assigned for each component present and participants classified as prefrail (2/5) or frail (≥3/5). The operational definitions followed the original Fried criteria: (1) low BMI defined as BMI <18.5 kg/m²; (2) exhaustion defined as a Functional Assessment of Chronic Illness Therapy-Fatigue score ≤30, indicating clinically meaningful fatigue; (3) low physical activity defined as <600 metabolic equivalent minute/week based on International Physical Activity Questionnaire-Short Form scoring guidelines; (4) slow gait speed defined as a 4-m walk speed <0.8 m/second; and (5) weakness defined as handgrip strength <26 kg for men and <18 kg for women. All measures were obtained remotely using mailed equipment and videoconference supervision. All frailty assessments were conducted under live videoconference supervision by trained research staff to ensure standardized administration.

#### Physical Function

We assessed physical function remotely using the Short Physical Performance Battery (SPPB) under the supervision (through videoconferencing) of study staff. Remote administration of the SPPB has previously been shown to demonstrate acceptable validity and reliability compared with in-person assessment when standardized instructions and camera positioning are used [18,19]. SPPB included 3 lower extremity measures completed in the following order: timed balance, gait speed, and chair stand. We evaluated timed balance under three conditions: (1) side-by-side stand with both feet placed on the ground for 10 seconds or when the participant steps out of position, (2) semitandem stand with the side of the heel of one foot touching the big toe of the other foot for 10 seconds or when the participant steps out of position, and (3) tandem stand with the heel of one foot touching the big toe of the other foot for 10 seconds or when the participant steps out of position. We evaluated chair stand performance under 2 conditions: participants performed a single chair stand, and then they were asked to perform 5 repeated chair stands as quickly as possible. The time taken to complete each task was recorded.

### Sample Size and Power Calculation

The original sample size calculation was based on detecting a between-group difference in frailty phenotype score at week 9, which was considered the primary early end point. Assuming a 2-sided α of 0.05, 80% power, 20% attrition, and a within-person correlation of 0.80 across repeated measures, 60 participants (30 per group) were estimated to be required to detect a standardized effect size (Cohen *d*) of 0.40. This effect size was selected based on prior exercise intervention studies demonstrating moderate improvements in frailty-related functional outcomes in clinical populations [20,21].

Owing to recruitment challenges in this specific posttransplant population, the final sample size was 32 participants (16 per group). Under the same assumptions, this sample size provided 80% power to detect a larger effect size of 0.50. Therefore, this study should be interpreted as an early-phase randomized trial with adequate power to detect moderate-to-large intervention effects but limited precision for smaller effects and generalizability.

### Statistical Analysis

We generated descriptive statistics for participants’ demographics, level of physical activity, and history of chronic health conditions. Assuming a 2-sided type I error of 0.05, intention-to-treat analyses were performed using all randomized participants. Changes in frailty index from baseline to week 9 and week 17 were evaluated using the Generalized Estimation Equation methods with 2 indicators of time, assuming a compound symmetry within-person correlation matrix. We included treatment by time interaction and tested its significance using a 2-*df* χ^2^ test. BMI, age, and race (non-Hispanic White vs other) were included as a priori covariates for adjustment. Additionally, between-group differences in outcome values at week 17 were computed, along with 95% CIs, and compared with differences at week 9.

### Ethical Considerations

The study protocol was approved by the COH Institutional Review Board (21406) and was conducted in accordance with institutional and national ethical standards for research involving human participants. All participants provided written informed consent electronically (DocuSign) prior to participation and were able to withdraw at any time without affecting their clinical care. Study data were deidentified and stored on secure institutional servers accessible only to authorized study personnel. Participants received a US $50 gift card as compensation for their time and participation. The trial was registered at ClinicalTrials.gov (NCT05142371) on October 29, 2021.

## Results

### Participant Flow and Retention

**Figure 1:**
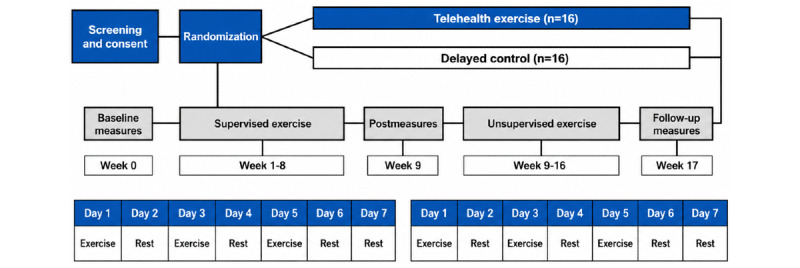
Study schema.

**Figure 2:**
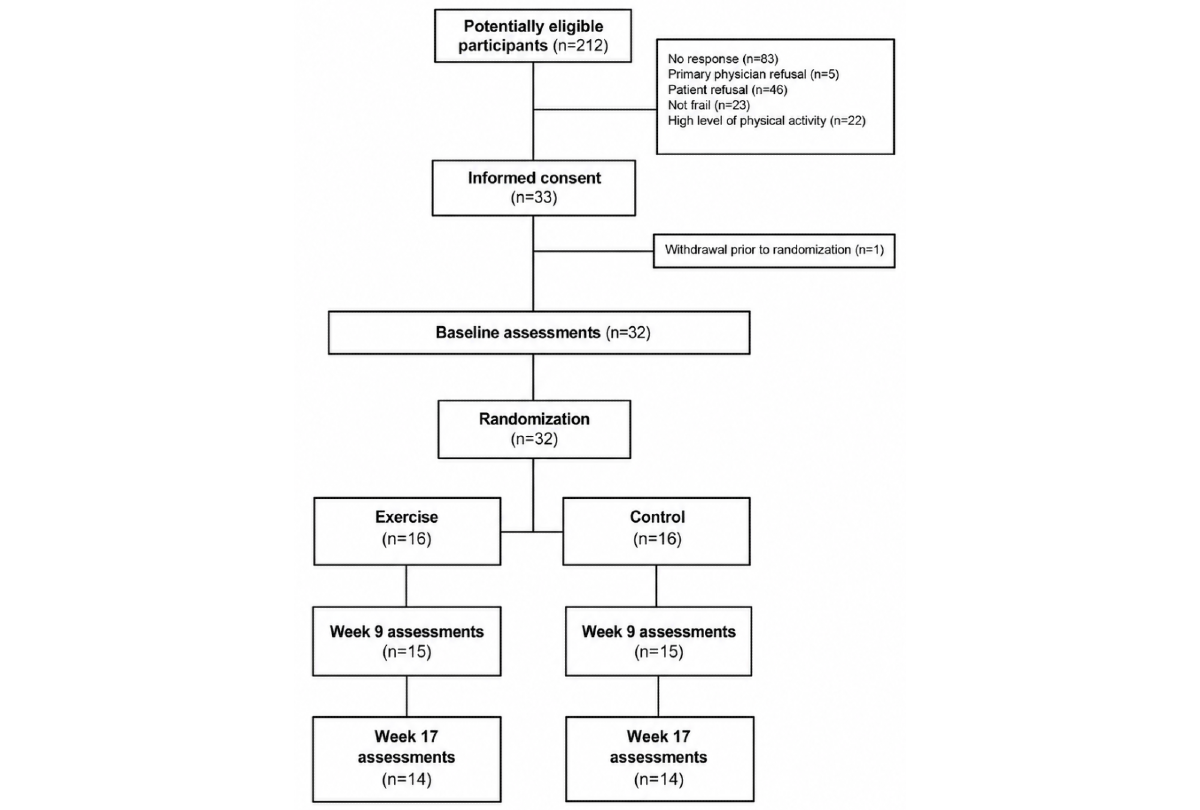
CONSORT (Consolidated Standards of Reporting Trials) diagram of mobile health–based exercise intervention.

A total of 212 potentially eligible patients with MM were identified in the long-term follow-up program following HCT at COH (Figure 1). Of the 129 successfully contacted patients with MM, 46 (36%) refused due to lack of interest, confidence in mHealth exercise settings, and time and 45 (35%) were deemed ineligible (Figure 2). Among the initial 33 prefrail or frail patients with MM who were enrolled, 1 individual withdrew from the study prior to randomization. This resulted in 32 patients randomized equally to either the mHealth exercise group (n=16) or the control group (n=16).

### Baseline Characteristics

Baseline characteristics are presented in Table 1. All participants had received high-dose melphalan conditioning (standard dose, 200 mg/m^2^), with appropriate dose reduction (140 mg/m^2^) for those with renal impairment (eg, creatinine clearance <40 mL/min), per standard of care. The mean age at study enrollment was 64.6 (SD 7.1) years. Of the 32 participants, 21 (66%) were female and 18 (56%) were non-Hispanic White. The mean time from HCT to study enrollment was 136 (SD 36.3) days. At baseline, 30 (94%) participants were classified as frail and 2 (6%) as prefrail according to the Fried phenotype criteria. The most common frailty characteristics were exhaustion (n=32, 100%) and low physical activity (n=30, 94%), followed by weakness (n=23, 72%), slow gait speed (n=19, 59%), and low BMI (n=1, 3%).

**Table 1:** Baseline participant characteristics (N=32).

			Total (N=32)		Exercise (n=16)		Control (n=16)
Age (years), mean (SD)			64.6 (7.1)		64.0 (6.9)		65.1 (7.5)
**Sex, n (%)**							
	Male	11 (34)		4 (25)		7 (44)	
	Female	21 (66)		12 (75)		9 (56)	
BMI (kg/m^2^), mean (SD)			26.1 (5.8)		27.3 (7.3)		24.9 (3.7)
**Race or ethnicity, n (%)**							
	Non-Hispanic White	18 (56)		9 (56)		9 (56)	
	Hispanic White	6 (19)		2 (13)		4 (25)	
	African American	5 (16)		3 (19)		2 (13)	
	Asian or Pacific Islander	3 (9)		2 (13)		1 (6)	
**Comorbidities, n (%)**							
	Hypertension	10 (31)		4 (25)		6 (38)	
	Diabetes	3 (9)		2 (13)		1 (6)	
	Dyslipidemia	8 (25)		5 (31)		3 (19)	
	Congestive heart failure	5 (16)		2 (13)		3 (19)	
**Frailty, n (%)**							
	Exhaustion	32 (100)		16 (100)		16 (100)	
	Low energy expenditure	30 (94)		15 (94)		15 (94)	
	Weakness	23 (72)		12 (75)		11 (69)	
	Slow gait speed	19 (59)		9 (56)		10 (63)	
	Low BMI	1 (3)		1 (6)		0 (0)	
Physical activity **(**MET^a^-min/week), mean (SD)			1441. 8 (812.2)		1490.9 (819.6)		1392.7 (828.4)

^a^MET: metabolic equivalent.

### Intervention Adherence and Safety

All participants (32/32, 100%) successfully completed remote physical function assessments prior to randomization and initiation of the intervention. At week 9, 2 (6%) participants were lost to follow-up, and an additional 2 (7%) were lost at week 17 (1 from each group at both time points). During the initial 8-week supervised phase, adherence to the 24 prescribed exercise sessions among participants in the intervention group was 85% (326/384 sessions). During the subsequent 8-week unsupervised phase, participants remained engaged with the mHealth platform and completed 78% (298/384 sessions) of the prescribed exercise sessions. No serious adverse events or unintended effects were reported during the intervention period.

### Primary Outcome: Frailty Phenotype

Overall, the mHealth exercise intervention resulted in a significant adjusted between-group reduction in frailty phenotype at week 9 (−0.92 points; 95% CI −1.38 to −0.46 ; *P*<.001), which was further sustained at week 17 (−1.14 points; 95% CI −1.66 to −0.62; Table 2). Descriptively, 63% (10/16) participants in the intervention group no longer met criteria for slow gait speed or low physical activity at both week 9 and week 17, whereas 88% (14/16) participants in the control group continued to meet these frailty components across the study period.

**Table 2:** Between-group differences in frailty and physical function outcomes at week 9 and week 17, estimated using generalized estimating equation models.

Outcomes			Baseline, mean (SD)		Week 9											Week 17						
					Mean (SD)			Between-group difference (95% CI)			*P* value			Mean (SD)				Between-group difference (95% CI)			*P* value	
**5-scale frailty phenotype**									−0.97 (−1.32 to −0.63)			<.001^a^							−1.19 (−1.72 to −0.66)			<.001^b^
	Exercise	3.94 (1.1)		3.07 (0.9)									2.79 (0.7)									
	Control	3.88 (1.1)		4.00 (0.9)									3.93 (0.9)									
**Chair stand performance**									−1.88 (−3.58 to −0.18)			.03^a^							−2.98 (−4.84 to −1.13)			.002^b^
	Exercise	16.8 (8.7)		13.6 (7.3)									12.0 (4.6)									
	Control	12.4 (3.1)		12.8 (3.7)									12.7 (3.6)									
**Gait speed (m/s)**									0.02 (−0.09 to 0.13)			.69							0.01 (−0.19 to 0.22)			.90
	Exercise	1.08 (0.5)		1.12 (0.5)									1.13 (0.3)									
	Control	1.01 (0.4)		1.07 (0.3)									1.12 (0.3)									
**Handgrip strength (dominant)**									0.71 (−1.54 to 2.97)			.54							2.43 (−0.57 to 5.44)			.11
	Exercise	28.1 (13.2)		29.6 (12.1)									32.6 (12.3)									
	Control	32.7 (13.1)		31.5 (12.8)									31.6 (11.1)									
**Handgrip strength (nondominant)**									1.78 (−0.77 to 4.35)			.17							1.19 (−2.06 to 4.46)			.47
	Exercise	24.8 (9.2)		27.5 (11.2)									28.4 (10.6)									
	Control	28.9 (13.1)		28.7 (13.4)									29.3 (12.2)									
**Short Physical Performance Battery**									0.35 (−0.20 to 0.92)			.21							0.64 (−0.14 to 1.42)			.11
	Exercise	10.3 (1.9)		10.6 (1.6)									10.8 (1.4)									
	Control	10.4 (1.3)		10.5 (1.5)									10.3 (1.5)									
**BMI**									−0.27 (−1.06 to 0.51)			.25							−0.45 (−1.38 to 0.48)			.35
	Exercise	27.3 (7.3)		27.7 (7.3)									28.2 (7.1)									
	Control	24.9 (3.7)		25.3 (3.5)									26.2 (3.2)									

^a^Between-group differences represent adjusted change estimates (exercise vs control) derived from generalized estimating equation models with 95% CIs based on intention-to-treat analysis.

^b^*P* values correspond to the treatment by time interaction terms from the same models.

### Secondary Outcomes: Physical Function

The mHealth exercise intervention resulted in a significant adjusted between-group improvement in chair stand performance at week 9 (−2.78 seconds; 95% CI −4.66 to −0.90) that was sustained at week 17 (−3.50 seconds; 95% CI −5.58 to −1.42; Table 2). The adjusted between-group difference in handgrip strength did not reach statistical significance. However, descriptively, dominant arm strength increased within the intervention group, with 50% (8/16) participants demonstrating an increase of >1 kg by week 9 that was maintained through week 17, whereas no consistent change was observed in the control group.

## Discussion

### Principal Findings

The findings of this study demonstrated the efficacy of an mHealth exercise intervention in improving frailty phenotype and physical function among patients with MM who had recently undergone autologous HCT. This study is among the first to evaluate a fully remote, supervised mHealth exercise intervention specifically targeting frailty reversal in patients with MM following transplantation. This indicates the potential for mHealth interventions to provide sustainable support and benefits for individuals with MM beyond their major cancer treatments. The observed improvements in chair stand performance highlight the potential of mHealth exercise to address key aspects of physical function in patients with MM. These outcomes are clinically meaningful, as each unit increase in chair stand score has been associated with a 21% improvement in survival among older adults [22].

### Comparison With Prior MM Trials

Randomized evidence supporting exercise and behavioral interventions in MM is emerging; however, prior trials have largely focused on behavioral coaching models rather than the delivery of structured, progressive exercise targeting functional recovery and frailty. The REAL-FITNESS trial randomized newly diagnosed patients to a World Health Organization–compliant physical activity prescription during induction therapy and demonstrated improvements in fatigue, depression, grip strength, comorbidity burden, and quality of life [23]. This study importantly established that promoting guideline-based physical activity is feasible and beneficial during treatment. However, the intervention primarily emphasized adherence to aerobic and resistance activity targets rather than delivering supervised, progressive exercise with quantified intensity, and the population did not include patients recovering from the physiological stress of transplantation where frailty risk is substantially heightened. Similarly, Larsen et al [24] conducted a randomized trial of a 10-week individualized exercise program in newly diagnosed patients with MM with careful adjustment for lytic bone disease. The trial demonstrated that exercise is safe in this population and may improve functional measures such as sit-to-stand performance and aerobic capacity. However, no clear between-group superiority was observed, and the study population consisted of patients earlier in their disease trajectory rather than individuals in the high-risk posttransplant recovery phase. Moreover, neither study used frailty as a primary outcome. In contrast, Banerjee et al [25] evaluated a digital life coaching intervention during stem cell transplantation and demonstrated that sustained patient engagement and patient-reported outcomes can be maintained through a fully remote support model even during the intensive peritransplant period. While this work demonstrated the potential of digital health platforms in MM care, the intervention focused on behavioral coaching and patient-reported outcomes rather than structured exercise delivery or objective functional end points.

This trial extends this literature in several clinically meaningful ways. First, it specifically targets the early recovery window following autologous transplantation and deliberately enrolls individuals who are prefrail or frail, a subgroup central to posttransplant morbidity but underrepresented in prior MM exercise trials. Second, the intervention delivers supervised, progressive exercise with explicit intensity and progression during the early phase, followed by a sustainability phase within the same mHealth ecosystem, rather than relying primarily on activity encouragement or coaching. Third, the study pairs this intervention with remote objective functional assessments and a frailty phenotype end point, directly evaluating frailty reversal rather than changes in activity volume or quality of life metrics alone. Collectively, these features position mHealth-supported exercise rehabilitation as a scalable strategy for functional recovery and frailty reversal in survivors of MM after transplantation, complementing and extending prior MM trials conducted in earlier disease phases and coaching-focused digital models.

### Interpretation and Potential Mechanisms

The observed adherence to the prescribed exercise sessions during both the supervised (326/384, 85%) and unsupervised phases of the intervention (298/384, 78%) is noteworthy. Despite the transition to unsupervised exercise sessions during the second 8-week phase, participants remained physically active and responsive to the prescribed exercise regimen. This indicates the feasibility and sustainability of mHealth exercise interventions in facilitating engagement in continuous exercise training among patients with MM, even in the absence of direct supervision. Developing a tailored exercise intervention within similar disease and treatment profiles may allow for health care professionals to address specific patient’s needs or challenges and enhance the effectiveness of rehabilitation strategies. One notable aspect of the findings from this study is the absence of significant improvement in gait speed following the intervention, despite the observed improvements in other measures of chair stand performance and frailty phenotype. Gait speed is widely used to assess functional capacity, and its assessment provides valuable insights into an individual’s ability to perform physical activities [26]. However, the discrepancy between self-reported improvements in gait and the lack of significant changes in objective measures of gait speed in our study may present an intriguing aspect of the study findings. While participants may have subjectively perceived improvements in their gait, as reflected in self-reported frailty phenotype assessments, these perceptions may not have translated into measurable changes in gait speed during objective assessments [27]. It is possible that participants experienced improvements in other aspects of gait performance, such as perceptions of effort or changes in overall mobility, that were not captured by the gait speed itself. Furthermore, variability in testing conditions, such as the testing environment and surface conditions, may have influenced gait speed measurements and introduced variability in the results.

### Limitations

While this study represents an important step forward in exploring the potential of mHealth exercise interventions to improve frailty phenotype and physical function in patients with MM, it is important to acknowledge certain limitations. First, the sample size was modest and drawn from a single center, which limits statistical precision and generalizability. Second, the control group did not receive an attention-matched intervention; therefore, nonspecific effects related to increased contact, accountability, or health-focused reminders cannot be excluded as contributors to the observed improvements. Third, eligibility required English proficiency and familiarity with telehealth technology, which may limit applicability to non-English speakers and individuals with lower digital literacy. Finally, although remote objective assessments were incorporated, some frailty components relied on self-report measures, which may introduce reporting bias.

### Conclusions

In conclusion, this study contributes to the growing body of evidence supporting the efficacy of mHealth exercise interventions in improving frailty phenotype and physical function among patients with MM. These findings have important implications for the development of accessible exercise interventions aimed at enhancing the functional capacity of individuals living with MM. Further research is needed to explore the long-term effects and potential mechanisms underlying the observed improvements, as well as to optimize the delivery and implementation of mHealth-based exercise programs in this population.

## Appendix

Multimedia Appendix 1 Representative structure of a supervised mobile health exercise session, including balance, strength, core stability, and functional mobility components, with repetitions-in-reserve–based rating of perceived exertion targets and progression rules.

Multimedia Appendix 2 CONSORT checklist.

## Abbreviations

COH: City of Hope
CONSORT: Consolidated Standards of Reporting Trials
HCT: hematopoietic cell transplantation
mHealth: mobile health
MM: multiple myeloma
RPE: rating of perceived exertion
SPPB: Short Physical Performance Battery

## Data Availability

The datasets generated and analyzed during this study are not publicly available due to institutional data protection policies but are available from the corresponding author on reasonable request and with appropriate institutional approvals.
