# Supplementary material for: mHealth-Supported Exercise Rehabilitation to Reverse Frailty After Autologous Transplantation in Multiple Myeloma: Randomized Controlled Trial
Source: JMIR Mhealth Uhealth. 2026 May 21;14:e87628. doi: 10.2196/87628 (PMC13237530; doi:10.2196/87628)
Supplement: Multimedia Appendix 1 [file mhealth_v14i1e87628_app1.docx]

**Multimedia Appendix 1.** Representative structure of a supervised mHealth exercise session including balance, strength, core stability, and functional mobility components with RIR-based RPE targets and progression rules.

| **Phase** | **Component** | **Example Exercises** | **Sets / Repetitions** | **Target RPE (RIR-based)** | **Progression Rule** |
| --- | --- | --- | --- | --- | --- |
| **Warm-up (5 min)** | Mobility & circulation | Marching in place, arm circles, gentle trunk rotations | Continuous 5 min | RPE 9–10 | Increase movement range as tolerated |
| **Balance** | Static & dynamic balance | Tandem stance, single-leg stance (with support), heel-to-toe walk | 3 × 30 sec each | RPE 10–11 | Narrow base of support, reduce hand support |
| **Strength (Lower body)** | Functional resistance | Sit-to-stand, wall squat, resistance band squat | 3 × 8–12 | **RPE 7–8** (2–3 reps in reserve) | Increase reps → sets → band tension |
| **Strength (Upper body)** | Resistance band training | Seated row, chest press, biceps curl | 3 × 10–12 | **RPE 7–8** | Increase band tension or reps |
| **Core stability** | Trunk control | Seated knee lift, bird-dog (modified), side hold | 3 × 10 | RPE 6–7 | Increase hold time or limb movement |
| **Functional mobility** | Integrated movement | Step-back lunge (supported), step-up, marching with band | 2–3 × 8 | RPE 7 | Reduce support, increase range |
| **Cool-down (5 min)** | Stretch & breathing | Hamstring stretch, chest opening, diaphragmatic breathing | Continuous 5 min | RPE <9 | — |
