# Supplementary material for: mHealth-Supported Exercise Rehabilitation to Reverse Frailty After Autologous Transplantation in Multiple Myeloma: Randomized Controlled Trial
Source: JMIR Mhealth Uhealth. 2026 May 21;14:e87628. doi: 10.2196/87628 (PMC13237530; doi:10.2196/87628)
Supplement: Multimedia Appendix 2 [file mhealth_v14i1e87628_app2.pdf]

CONSORT-EHEALTH (V 1.6.1) - Submission/Publication Form

The CONSORT-EHEALTH checklist is intended for authors of randomized trials evaluating web-based and Internet-based applications/interventions, including mobile interventions, electronic games (incl multiplayer games), social media, certain telehealth applications, and other interactive and/or networked electronic applications. Some of the items (e.g. all subitems under item 5 - description of the intervention) may also be applicable for other study designs.

The goal of the CONSORT EHEALTH checklist and guideline is to be
a) a guide for reporting for authors of RCTs,
b) to form a basis for appraisal of an ehealth trial (in terms of validity)

CONSORT-EHEALTH items/subitems are MANDATORY reporting items for studies published in the Journal of Medical Internet Research and other journals / scientific societies endorsing the checklist.

Items numbered 1., 2., 3., 4a., 4b etc are original CONSORT or CONSORT-NPT (non-pharmacologic treatment) items.
Items with Roman numerals (i., ii, iii, iv etc.) are CONSORT-EHEALTH extensions/clarifications.

As the CONSORT-EHEALTH checklist is still considered in a formative stage, we would ask that you also RATE ON A SCALE OF 1-5 how important/useful you feel each item is FOR THE PURPOSE OF THE CHECKLIST and reporting guideline (optional).

Mandatory reporting items are marked with a red *.
In the textboxes, either copy & paste the relevant sections from your manuscript into this form - please include any quotes from your manuscript in QUOTATION MARKS,
or answer directly by providing additional information not in the manuscript, or elaborating on why the item was not relevant for this study.

YOUR ANSWERS WILL BE PUBLISHED AS A SUPPLEMENTARY FILE TO YOUR PUBLICATION IN JMIR AND ARE CONSIDERED PART OF YOUR PUBLICATION (IF ACCEPTED).
Please fill in these questions diligently. Information will not be copyedited, so please use proper spelling and grammar, use correct capitalization, and avoid abbreviations.

DO NOT FORGET TO SAVE AS PDF _AND_ CLICK THE SUBMIT BUTTON SO YOUR ANSWERS ARE IN OUR DATABASE !!!

Citation Suggestion (if you append the pdf as Appendix we suggest to cite this paper in the caption):
Eysenbach G, CONSORT-EHEALTH Group
CONSORT-EHEALTH: Improving and Standardizing Evaluation Reports of Web-based and Mobile Health Interventions
J Med Internet Res 2011;13(4):e126
URL: http://www.jmir.org/2011/4/e126/
doi: 10.2196/jmir.1923
PMID: 22209829

kulee2533@gmail.com Switch account
Not shared
Resubmit to save

* Indicates required question

Your name *
First Last

Kyuwan Lee

Primary Affiliation (short), City, Country *
University of Toronto, Toronto, Canada

Ewha Womans University

Your e-mail address *
abc@gmail.com

kyuwanlee@ewha.ac.kr

Title of your manuscript *
Provide the (draft) title of your manuscript.

mHealth-Supported Exercise Rehabilitation to Reverse Frailty After Autologous Transplantation in Multiple Myeloma: A Randomized Controlled Trial

Name of your App/Software/Intervention *
If there is a short and a long/alternate name, write the short name first and add the long name in brackets.

mHealth-supported tele-exercise and remote n

Evaluated Version (if any)
e.g. "V1", "Release 2017-03-01", "Version 2.0.27913"

Your answer

You're editing your response. Sharing this URL allows others to also edit your response. FILL OUT A NEW RESPONSE

Language(s) *

What language is the intervention/app in? If multiple languages are available, separate by comma (e.g. "English, French")

English

URL of your Intervention Website or App

e.g. a direct link to the mobile app on app in appstore (itunes, Google Play), or URL of the website. If the intervention is a DVD or hardware, you can also link to an Amazon page.

Your answer

URL of an image/screenshot (optional)

Your answer

Accessibility *

Can an enduser access the intervention presently?

- ○ access is free and open
- ◉ access only for special usergroups, not open
- ○ access is open to everyone, but requires payment/subscription/in-app purchases
- ○ app/intervention no longer accessible
- ○ Other:

Primary Medical Indication/Disease/Condition *

e.g. "Stress", "Diabetes", or define the target group in brackets after the condition, e.g. "Autism (Parents of children with)", "Alzheimers (Informal Caregivers of)"

Multiple Myeloma

Primary Outcomes measured in trial *

comma-separated list of primary outcomes reported in the trial

Frailty

Secondary/other outcomes

Are there any other outcomes the intervention is expected to affect?

Chair stand time, handgrip strength, gait speed

Recommended "Dose" *

What do the instructions for users say on how often the app should be used?

- ◉ Approximately Daily
- ○ Approximately Weekly
- ○ Approximately Monthly
- ○ Approximately Yearly
- ○ "as needed"
- ○ Other:

Approx. Percentage of Users (starters) still using the app as recommended after 3 months *

- ◉ unknown / not evaluated
- ○ 0-10%
- ○ 11-20%
- ○ 21-30%
- ○ 31-40%
- ○ 41-50%
- ○ 51-60%
- ○ 61-70%
- ○ 71%-80%
- ○ 81-90%
- ○ 91-100%
- ○ Other:

You're editing your response. Sharing this URL allows others to also edit your response. FILL OUT A NEW RESPONSE

Overall, was the app/intervention effective? *

- (selected) yes: all primary outcomes were significantly better in intervention group vs control
- partly: SOME primary outcomes were significantly better in intervention group vs control
- no statistically significant difference between control and intervention
- potentially harmful: control was significantly better than intervention in one or more outcomes
- inconclusive: more research is needed
- Other:

Article Preparation Status/Stage *

At which stage in your article preparation are you currently (at the time you fill in this form)

- not submitted yet - in early draft status
- not submitted yet - in late draft status, just before submission
- submitted to a journal but not reviewed yet
- (selected) submitted to a journal and after receiving initial reviewer comments
- submitted to a journal and accepted, but not published yet
- published
- Other:

Journal *

If you already know where you will submit this paper (or if it is already submitted), please provide the journal name (if it is not JMIR, provide the journal name under "other")

- not submitted yet / unclear where I will submit this
- Journal of Medical Internet Research (JMIR)
- (selected) JMIR mHealth and UHealth
- JMIR Serious Games
- JMIR Mental Health
- JMIR Public Health
- JMIR Formative Research
- Other JMIR sister journal
- Other:

Is this a full powered effectiveness trial or a pilot/feasibility trial? *

- (selected) Pilot/feasibility
- Fully powered

Manuscript tracking number *

If this is a JMIR submission, please provide the manuscript tracking number under "other" (The ms tracking number can be found in the submission acknowledgement email, or when you login as author in JMIR. If the paper is already published in JMIR, then the ms tracking number is the four-digit number at the end of the DOI, to be found at the bottom of each published article in JMIR)

- no ms number (yet) / not (yet) submitted to / published in JMIR
- (selected) Other: 87628

TITLE AND ABSTRACT

1a) TITLE: Identification as a randomized trial in the title

1a) Does your paper address CONSORT item 1a? *

I.e does the title contain the phrase "Randomized Controlled Trial"? (if not, explain the reason under "other")

- (selected) yes
- Other:

You're editing your response. Sharing this URL allows others to also edit your response. FILL OUT A NEW RESPONSE

1a-i) Identify the mode of delivery in the title

Identify the mode of delivery. Preferably use “web-based” and/or “mobile” and/or “electronic game” in the title. Avoid ambiguous terms like “online”, “virtual”, “interactive”. Use “Internet-based” only if Intervention includes non-web-based Internet components (e.g. email), use “computer-based” or “electronic” only if offline products are used. Use “virtual” only in the context of “virtual reality” (3-D worlds). Use “online” only in the context of “online support groups”. Complement or substitute product names with broader terms for the class of products (such as “mobile” or “smart phone” instead of “iphone”), especially if the application runs on different platforms.

	1	2	3	4	5	
subitem not at all important	○	○	○	○	●	essential

Clear selection

Does your paper address subitem 1a-i? *

Copy and paste relevant sections from manuscript title (include quotes in quotation marks "like this" to indicate direct quotes from your manuscript), or elaborate on this item by providing additional information not in the ms, or briefly explain why the item is not applicable/relevant for your study

mHealth-Supported Exercise Rehabilitation to Reverse Frailty After Autologous Transplantation in Multiple Myeloma: A Randomized Controlled Trial

1a-ii) Non-web-based components or important co-interventions in title

Mention non-web-based components or important co-interventions in title, if any (e.g., “with telephone support”).

	1	2	3	4	5	
subitem not at all important	○	○	●	○	○	essential

Clear selection

Does your paper address subitem 1a-ii?

Copy and paste relevant sections from manuscript title (include quotes in quotation marks "like this" to indicate direct quotes from your manuscript), or elaborate on this item by providing additional information not in the ms, or briefly explain why the item is not applicable/relevant for your study

mHealth-Supported Exercise Rehabilitation to Reverse Frailty After Autologous Transplantation in Multiple Myeloma: A Randomized Controlled Trial

1a-iii) Primary condition or target group in the title

Mention primary condition or target group in the title, if any (e.g., “for children with Type I Diabetes”) Example: A Web-based and Mobile Intervention with Telephone Support for Children with Type I Diabetes: Randomized Controlled Trial

	1	2	3	4	5	
subitem not at all important	○	○	○	○	●	essential

Clear selection

Does your paper address subitem 1a-iii? *

Copy and paste relevant sections from manuscript title (include quotes in quotation marks "like this" to indicate direct quotes from your manuscript), or elaborate on this item by providing additional information not in the ms, or briefly explain why the item is not applicable/relevant for your study

mHealth-Supported Exercise Rehabilitation to Reverse Frailty After Autologous Transplantation in Multiple Myeloma: A Randomized Controlled Trial

1b) ABSTRACT: Structured summary of trial design, methods, results, and conclusions

NPT extension: Description of experimental treatment, comparator, care providers, centers, and blinding status.

1b-i) Key features/functionalities/components of the intervention and comparator in the METHODS section of the ABSTRACT

Mention key features/functionalities/components of the intervention and comparator in the abstract. If possible, also mention theories and principles used for designing the site. Keep in mind the needs of systematic reviewers and indexers by including important synonyms. (Note: Only report in the abstract what the main paper is reporting. If this information is missing from the main body of text, consider adding it)

	1	2	3	4	5	
subitem not at all important	○	○	○	○	●	essential

Clear selection

You're editing your response. Sharing this URL allows others to also edit your response.

FILL OUT A NEW RESPONSE

Does your paper address subitem 1b-i? *

Copy and paste relevant sections from the manuscript abstract (include quotes in quotation marks "like this" to indicate direct quotes from your manuscript), or elaborate on this item by providing additional information not in the ms, or briefly explain why the item is not applicable/relevant for your study

에 넣을 답변 (따옴표 포함해서 그대로)

“The intervention comprised 8 weeks of supervised tele-exercise (3 sessions/week, ~50 minutes/session) delivered via videoconferencing, followed by 8 weeks of home-based exercise supported through the same mHealth platform with remote monitoring and feedback.”

1b-ii) Level of human involvement in the METHODS section of the ABSTRACT

Clarify the level of human involvement in the abstract, e.g., use phrases like “fully automated” vs. “therapist/nurse/care provider/physician-assisted” (mention number and expertise of providers involved, if any). (Note: Only report in the abstract what the main paper is reporting. If this information is missing from the main body of text, consider adding it)

	1	2	3	4	5	
subitem not at all important	○	○	○	○	◉	essential

Clear selection

Does your paper address subitem 1b-ii?

Copy and paste relevant sections from the manuscript abstract (include quotes in quotation marks "like this" to indicate direct quotes from your manuscript), or elaborate on this item by providing additional information not in the ms, or briefly explain why the item is not applicable/relevant for your study

Your answer

1b-iii) Open vs. closed, web-based (self-assessment) vs. face-to-face assessments in the METHODS section of the ABSTRACT

Mention how participants were recruited (online vs. offline), e.g., from an open access website or from a clinic or a closed online user group (closed usergroup trial), and clarify if this was a purely web-based trial, or there were face-to-face components (as part of the intervention or for assessment). Clearly say if outcomes were self-assessed through questionnaires (as common in web-based trials). Note: In traditional offline trials, an open trial (open-label trial) is a type of clinical trial in which both the researchers and participants know which treatment is being administered. To avoid confusion, use “blinded” or “unblinded” to indicated the level of blinding instead of “open”, as “open” in web-based trials usually refers to “open access” (i.e. participants can self-enrol). (Note: Only report in the abstract what the main paper is reporting. If this information is missing from the main body of text, consider adding it)

	1	2	3	4	5	
subitem not at all important	○	○	○	○	◉	essential

Clear selection

Does your paper address subitem 1b-iii?

Copy and paste relevant sections from the manuscript abstract (include quotes in quotation marks "like this" to indicate direct quotes from your manuscript), or elaborate on this item by providing additional information not in the ms, or briefly explain why the item is not applicable/relevant for your study

“The intervention comprised 8 weeks of supervised tele-exercise delivered through videoconferencing by exercise specialists (3 sessions/week, ~50 minutes/session), followed by 8 weeks of remotely monitored home-based exercise using the same mHealth platform.”

1b-iv) RESULTS section in abstract must contain use data

Report number of participants enrolled/assessed in each group, the use/uptake of the intervention (e.g., attrition/adherence metrics, use over time, number of logins etc.), in addition to primary/secondary outcomes. (Note: Only report in the abstract what the main paper is reporting. If this information is missing from the main body of text, consider adding it)

	1	2	3	4	5	
subitem not at all important	○	○	○	○	◉	essential

Clear selection

Does your paper address subitem 1b-iv?

Copy and paste relevant sections from the manuscript abstract (include quotes in quotation marks "like this" to indicate direct quotes from your manuscript), or elaborate on this item by providing additional information not in the ms, or briefly explain why the item is not applicable/relevant for your study

All participants in the exercise group completed the 8-week supervised tele-exercise phase, and 14 of 16 (87.5%) continued the home-based phase through week 17. No serious adverse events occurred.

You're editing your response. Sharing this URL allows others to also edit your response.

FILL OUT A NEW RESPONSE

1b-v) CONCLUSIONS/DISCUSSION in abstract for negative trials

Conclusions/Discussions in abstract for negative trials: Discuss the primary outcome - if the trial is negative (primary outcome not changed), and the intervention was not used, discuss whether negative results are attributable to lack of uptake and discuss reasons. (Note: Only report in the abstract what the main paper is reporting. If this information is missing from the main body of text, consider adding it)

	1	2	3	4	5	
subitem not at all important	◉	○	○	○	○	essential

Clear selection

Does your paper address subitem 1b-v?

Copy and paste relevant sections from the manuscript abstract (include quotes in quotation marks "like this" to indicate direct quotes from your manuscript), or elaborate on this item by providing additional information not in the ms, or briefly explain why the item is not applicable/relevant for your study

Not applicable. The primary outcome showed a statistically significant improvement in the intervention group, and intervention adherence was high. Therefore, this item for negative trials is not applicable to this study.

INTRODUCTION

2a) In INTRODUCTION: Scientific background and explanation of rationale

2a-i) Problem and the type of system/solution

Describe the problem and the type of system/solution that is object of the study: intended as stand-alone intervention vs. incorporated in broader health care program? Intended for a particular patient population? Goals of the intervention, e.g., being more cost-effective to other interventions, replace or complement other solutions? (Note: Details about the intervention are provided in "Methods" under 5)

	1	2	3	4	5	
subitem not at all important	○	○	○	○	◉	essential

Clear selection

Does your paper address subitem 2a-i? *

Copy and paste relevant sections from the manuscript (include quotes in quotation marks "like this" to indicate direct quotes from your manuscript), or elaborate on this item by providing additional information not in the ms, or briefly explain why the item is not applicable/relevant for your study

"Patients with multiple myeloma undergoing autologous hematopoietic cell transplantation frequently experience profound declines in physical function and increased frailty, yet structured rehabilitation during this vulnerable recovery period is rarely implemented due to logistical barriers, symptom burden, and limited access to supervised exercise programs. mHealth-supported exercise interventions offer a potential solution by enabling supervised, structured rehabilitation to be delivered remotely during early post-transplant recovery. This approach is intended to complement standard clinical care by addressing functional decline and frailty through accessible, home-based exercise rehabilitation in a high-risk population."

2a-ii) Scientific background, rationale: What is known about the (type of) system

Scientific background, rationale: What is known about the (type of) system that is the object of the study (be sure to discuss the use of similar systems for other conditions/diagnoses, if appropiate), motivation for the study, i.e. what are the reasons for and what is the context for this specific study, from which stakeholder viewpoint is the study performed, potential impact of findings [2]. Briefly justify the choice of the comparator.

	1	2	3	4	5	
subitem not at all important	○	○	○	○	◉	essential

Clear selection

You're editing your response. Sharing this URL allows others to also edit your response.

FILL OUT A NEW RESPONSE

Does your paper address subitem 2a-ii? *

Copy and paste relevant sections from the manuscript (include quotes in quotation marks "like this" to indicate direct quotes from your manuscript), or elaborate on this item by providing additional information not in the ms, or briefly explain why the item is not applicable/relevant for your study

"mHealth and tele-exercise systems have been increasingly used in oncology, cardiometabolic disease, and geriatric rehabilitation to overcome barriers to supervised exercise, improve adherence, and enable remote functional monitoring. However, their application in hematologic malignancies, particularly during early recovery after autologous transplantation, remains limited. Prior exercise trials in multiple myeloma have primarily focused on newly diagnosed patients during induction therapy or on behavioral coaching approaches that target health behaviors rather than structured, progressive exercise training. For example, the REAL-FITNESS trial demonstrated feasibility and benefits of WHO-aligned physical activity promotion during induction therapy, Larsen et al showed safety of individualized exercise considering bone disease, and Banerjee et al demonstrated feasibility of digital life coaching during transplantation. These studies support the feasibility of remote or structured activity promotion in MM but do not directly address frailty-targeted, progressive exercise rehabilitation during the early post-transplant recovery window.

Therefore, this study was designed to evaluate whether a structured mHealth-supported exercise rehabilitation program could complement standard post-transplant care by targeting frailty reversal and functional recovery in a high-risk population. Usual care was selected as the comparator to reflect real-world clinical practice in which no structured rehabilitation is routinely provided after transplantation."

2b) In INTRODUCTION: Specific objectives or hypotheses

Does your paper address CONSORT subitem 2b? *

Copy and paste relevant sections from the manuscript (include quotes in quotation marks "like this" to indicate direct quotes from your manuscript), or elaborate on this item by providing additional information not in the ms, or briefly explain why the item is not applicable/relevant for your study

"The aim of this study was to evaluate whether a 16-week mHealth-supported exercise rehabilitation program could improve frailty status and physical function in multiple myeloma survivors within 180 days after autologous hematopoietic cell transplantation. We hypothesized that participants receiving the mHealth-supported exercise intervention would demonstrate greater improvements in frailty phenotype and lower-extremity functional performance compared with usual care."

METHODS

3a) Description of trial design (such as parallel, factorial) including allocation ratio

Does your paper address CONSORT subitem 3a? *

Copy and paste relevant sections from the manuscript (include quotes in quotation marks "like this" to indicate direct quotes from your manuscript), or elaborate on this item by providing additional information not in the ms, or briefly explain why the item is not applicable/relevant for your study

"This study was a single-center, parallel-group randomized controlled trial with a 1:1 allocation ratio comparing an mHealth-supported exercise intervention with usual care."

3b) Important changes to methods after trial commencement (such as eligibility criteria), with reasons

Does your paper address CONSORT subitem 3b? *

Copy and paste relevant sections from the manuscript (include quotes in quotation marks "like this" to indicate direct quotes from your manuscript), or elaborate on this item by providing additional information not in the ms, or briefly explain why the item is not applicable/relevant for your study

"No changes to methods, eligibility criteria, or outcome measures were made after trial commencement."

3b-i) Bug fixes, Downtimes, Content Changes

Bug fixes, Downtimes, Content Changes: ehealth systems are often dynamic systems. A description of changes to methods therefore also includes important changes made on the intervention or comparator during the trial (e.g., major bug fixes or changes in the functionality or content) (5-iii) and other "unexpected events" that may have influenced study design such as staff changes, system failures/downtimes, etc. [2].

	1	2	3	4	5	
subitem not at all important	○	○	○	●	○	essential

Clear selection

Does your paper address subitem 3b-i?

Copy and paste relevant sections from the manuscript (include quotes in quotation marks "like this" to indicate direct quotes from your manuscript), or elaborate on this item by providing additional information not in the ms, or briefly explain why the item is not applicable/relevant for your study

"No bug fixes, system downtimes, content changes, or modifications to the mHealth platform or intervention procedures occurred during the trial period."

You're editing your response. Sharing this URL allows others to also edit your response.

FILL OUT A NEW RESPONSE

4a) Eligibility criteria for participants

Does your paper address CONSORT subitem 4a? *

Copy and paste relevant sections from the manuscript (include quotes in quotation marks "like this" to indicate direct quotes from your manuscript), or elaborate on this item by providing additional information not in the ms, or briefly explain why the item is not applicable/relevant for your study

"Eligibility criteria included: (1) diagnosis of multiple myeloma; (2) >18 years of age; (3) pre-frail or frail status based on Fried criteria (2/5 indices = pre-frail; ≥3/5 = frail); (4) <180 days post-autologous HCT; (5) physically able and willing to complete all study procedures; and (6) English-speaking. Exclusion criteria included clinically significant/active cardiovascular disease, contraindications to exercise (eg, acute infectious disease or unstable bone lesions), recent injury within 6 months limiting rigorous exercise, and current participation in regular structured exercise (>60 minutes/week)."

4a-i) Computer / Internet literacy

Computer / Internet literacy is often an implicit "de facto" eligibility criterion - this should be explicitly clarified.

	1	2	3	4	5	
subitem not at all important	○	○	○	○	◉	essential

Clear selection

Does your paper address subitem 4a-i?

Copy and paste relevant sections from the manuscript (include quotes in quotation marks "like this" to indicate direct quotes from your manuscript), or elaborate on this item by providing additional information not in the ms, or briefly explain why the item is not applicable/relevant for your study

"Because all study procedures were conducted remotely (screening, consent, assessments, and intervention delivery), participants were required to have basic access to and ability to use an internet-enabled device capable of videoconferencing and engaging with the mHealth platform. English-speaking participation was required, and participants needed to be able to complete telehealth-based procedures, including joining videoconference visits for supervised sessions and remote assessments, and using the mHealth platform for exercise delivery and adherence tracking."

4a-ii) Open vs. closed, web-based vs. face-to-face assessments:

Open vs. closed, web-based vs. face-to-face assessments: Mention how participants were recruited (online vs. offline), e.g., from an open access website or from a clinic, and clarify if this was a purely web-based trial, or there were face-to-face components (as part of the intervention or for assessment), i.e., to what degree got the study team to know the participant. In online-only trials, clarify if participants were quasi-anonymous and whether having multiple identities was possible or whether technical or logistical measures (e.g., cookies, email confirmation, phone calls) were used to detect/prevent these.

	1	2	3	4	5	
subitem not at all important	○	○	○	○	◉	essential

Clear selection

Does your paper address subitem 4a-ii? *

Copy and paste relevant sections from the manuscript (include quotes in quotation marks "like this" to indicate direct quotes from your manuscript), or elaborate on this item by providing additional information not in the ms, or briefly explain why the item is not applicable/relevant for your study

"This was a closed user-group trial with offline clinical recruitment at a single center (City of Hope). Prescreening and outreach occurred via the long-term follow-up program and telephone contact, and frailty status was confirmed during the baseline remote assessment prior to randomization. Participants were known to the study team; there was no anonymous or open online self-enrollment."

4a-iii) Information giving during recruitment

Information given during recruitment. Specify how participants were briefed for recruitment and in the informed consent procedures (e.g., publish the informed consent documentation as appendix, see also item X26), as this information may have an effect on user self-selection, user expectation and may also bias results.

	1	2	3	4	5	
subitem not at all important	○	○	○	○	◉	essential

Clear selection

Does your paper address subitem 4a-iii?

Copy and paste relevant sections from the manuscript (include quotes in quotation marks "like this" to indicate direct quotes from your manuscript), or elaborate on this item by providing additional information not in the ms, or briefly explain why the item is not applicable/relevant for your study

"Eligible patients received detailed study information during telephone discussions with trained research staff and then provided electronic informed consent using a web-based consent form (DocuSign™). Participants were informed that they would be randomized to either an mHealth-supported exercise intervention or usual care and that all assessments would occur remotely under videoconference supervision using mailed equipment."

You're editing your response. Sharing this URL allows others to also edit your response.

FILL OUT A NEW RESPONSE

4b) Settings and locations where the data were collected

Does your paper address CONSORT subitem 4b? *

Copy and paste relevant sections from the manuscript (include quotes in quotation marks "like this" to indicate direct quotes from your manuscript), or elaborate on this item by providing additional information not in the ms, or briefly explain why the item is not applicable/relevant for your study

"This single-center trial was conducted at City of Hope; however, screening, enrollment, intervention delivery, and data collection were performed using an mHealth-based approach, eliminating the need for participants to be assessed at a center. Assessments were conducted remotely at baseline (week 0), week 9, and week 17 under live videoconference supervision."

4b-i) Report if outcomes were (self-)assessed through online questionnaires

Clearly report if outcomes were (self-)assessed through online questionnaires (as common in web-based trials) or otherwise.

	1	2	3	4	5	
subitem not at all important	○	○	○	●	○	essential

Clear selection

Does your paper address subitem 4b-i? *

Copy and paste relevant sections from the manuscript (include quotes in quotation marks "like this" to indicate direct quotes from your manuscript), or elaborate on this item by providing additional information not in the ms, or briefly explain why the item is not applicable/relevant for your study

"Outcomes were not self-assessed via open online questionnaires. Frailty phenotype components and physical function measures were obtained remotely using mailed measurement devices and live videoconference supervision by trained research staff to ensure standardized administration."

4b-ii) Report how institutional affiliations are displayed

Report how institutional affiliations are displayed to potential participants [on ehealth media], as affiliations with prestigious hospitals or universities may affect volunteer rates, use, and reactions with regards to an intervention.(Not a required item – describe only if this may bias results)

	1	2	3	4	5	
subitem not at all important	○	●	○	○	○	essential

Clear selection

Does your paper address subitem 4b-ii?

Copy and paste relevant sections from the manuscript (include quotes in quotation marks "like this" to indicate direct quotes from your manuscript), or elaborate on this item by providing additional information not in the ms, or briefly explain why the item is not applicable/relevant for your study

"Recruitment occurred through City of Hope clinical follow-up pathways and direct outreach, and all study procedures were represented as a City of Hope research study delivered by the study team through a commercially available mHealth platform."

5) The interventions for each group with sufficient details to allow replication, including how and when they were actually administered

5-i) Mention names, credential, affiliations of the developers, sponsors, and owners

Mention names, credential, affiliations of the developers, sponsors, and owners [6] (if authors/evaluators are owners or developer of the software, this needs to be declared in a "Conflict of interest" section or mentioned elsewhere in the manuscript).

	1	2	3	4	5	
subitem not at all important	○	○	○	○	●	essential

Clear selection

Does your paper address subitem 5-i?

Copy and paste relevant sections from the manuscript (include quotes in quotation marks "like this" to indicate direct quotes from your manuscript), or elaborate on this item by providing additional information not in the ms, or briefly explain why the item is not applicable/relevant for your study

"We used a commercially available mHealth exercise platform (Moterum Technologies, Salt Lake City, USA) that offers exercise videos, physical activity monitoring, and automatic data collection from wearable devices. The study team developed the exercise prescription, progression rules, and remote assessment workflow; the investigators were not owners of the commercial platform."

You're editing your response. Sharing this URL allows others to also edit your response.

FILL OUT A NEW RESPONSE

5-ii) Describe the history/development process

Describe the history/development process of the application and previous formative evaluations (e.g., focus groups, usability testing), as these will have an impact on adoption/use rates and help with interpreting results.

	1	2	3	4	5	
subitem not at all important	○	○	○	○	◉	essential

Clear selection

Does your paper address subitem 5-ii?

Copy and paste relevant sections from the manuscript (include quotes in quotation marks "like this" to indicate direct quotes from your manuscript), or elaborate on this item by providing additional information not in the ms, or briefly explain why the item is not applicable/relevant for your study

"The intervention procedures and remote assessment workflow were based on established exercise rehabilitation principles and were aligned with the previously published trial protocol (Telehealth exercise to Improve Physical function and frailty in patients with multiple myeloma treated with autologous hematopoietic stem cell transplantation [TIPS]). Details of the intervention strategies have been previously published."

5-iii) Revisions and updating

Revisions and updating. Clearly mention the date and/or version number of the application/intervention (and comparator, if applicable) evaluated, or describe whether the intervention underwent major changes during the evaluation process, or whether the development and/or content was "frozen" during the trial. Describe dynamic components such as news feeds or changing content which may have an impact on the replicability of the intervention (for unexpected events see item 3b).

	1	2	3	4	5	
subitem not at all important	○	○	○	○	◉	essential

Clear selection

Does your paper address subitem 5-iii?

Copy and paste relevant sections from the manuscript (include quotes in quotation marks "like this" to indicate direct quotes from your manuscript), or elaborate on this item by providing additional information not in the ms, or briefly explain why the item is not applicable/relevant for your study

"The commercial platform and intervention procedures were not modified during the trial, and no major updates, downtimes, or content changes necessitating protocol alterations occurred during the evaluation period."

5-iv) Quality assurance methods

Provide information on quality assurance methods to ensure accuracy and quality of information provided [1], if applicable.

	1	2	3	4	5	
subitem not at all important	○	○	○	○	◉	essential

Clear selection

Does your paper address subitem 5-iv?

Copy and paste relevant sections from the manuscript (include quotes in quotation marks "like this" to indicate direct quotes from your manuscript), or elaborate on this item by providing additional information not in the ms, or briefly explain why the item is not applicable/relevant for your study

"Quality assurance included standardized videoconference-supervised assessment procedures, trained research staff oversight, and standardized symptom-guided progression rules. Adherence was tracked on the platform, and exercise sessions could be rescheduled or made up if participants were unable to attend as planned."

5-v) Ensure replicability by publishing the source code, and/or providing screenshots/screen-capture video, and/or providing flowcharts of the algorithms used

Ensure replicability by publishing the source code, and/or providing screenshots/screen-capture video, and/or providing flowcharts of the algorithms used. Replicability (i.e., other researchers should in principle be able to replicate the study) is a hallmark of scientific reporting.

	1	2	3	4	5	
subitem not at all important	○	○	○	○	◉	essential

Clear selection

You're editing your response. Sharing this URL allows others to also edit your response. FILL OUT A NEW RESPONSE

Does your paper address subitem 5-v?

Copy and paste relevant sections from the manuscript (include quotes in quotation marks "like this" to indicate direct quotes from your manuscript), or elaborate on this item by providing additional information not in the ms, or briefly explain why the item is not applicable/relevant for your study

"Replicability was supported through explicit reporting of intervention dose (24 supervised sessions over 8 weeks, 3 sessions/week, ~50 minutes/session), intensity prescription using RIR-based RPE targets, symptom-guided progression rules, and mailed equipment for remote measurement. A representative example of a supervised exercise session structure is provided in Multimedia Appendix 1 to facilitate reproducibility of the intervention."

5-vi) Digital preservation

Digital preservation: Provide the URL of the application, but as the intervention is likely to change or disappear over the course of the years; also make sure the intervention is archived (Internet Archive, webcitation.org, and/or publishing the source code or screenshots/videos alongside the article). As pages behind login screens cannot be archived, consider creating demo pages which are accessible without login.

	1	2	3	4	5	
subitem not at all important	○	○	○	○	◉	essential

Clear selection

Does your paper address subitem 5-vi?

Copy and paste relevant sections from the manuscript (include quotes in quotation marks "like this" to indicate direct quotes from your manuscript), or elaborate on this item by providing additional information not in the ms, or briefly explain why the item is not applicable/relevant for your study

"Because the intervention was delivered through a commercial platform requiring participant access, public web archiving of login-protected content is not feasible. However, intervention procedures, session structure, and progression rules are preserved through the published protocol and Multimedia Appendix materials."

5-vii) Access

Access: Describe how participants accessed the application, in what setting/context, if they had to pay (or were paid) or not, whether they had to be a member of specific group. If known, describe how participants obtained "access to the platform and Internet" [1]. To ensure access for editors/reviewers/readers, consider to provide a "backdoor" login account or demo mode for reviewers/readers to explore the application (also important for archiving purposes, see vi).

	1	2	3	4	5	
subitem not at all important	○	○	○	○	◉	essential

Clear selection

Does your paper address subitem 5-vii? *

Copy and paste relevant sections from the manuscript (include quotes in quotation marks "like this" to indicate direct quotes from your manuscript), or elaborate on this item by providing additional information not in the ms, or briefly explain why the item is not applicable/relevant for your study

"Participants accessed the intervention through the commercially available mHealth platform and videoconferencing. Exercise equipment (resistance bands, loop bands, and exercise mats) and assessment tools (gait sensors, hand dynamometer, measuring tape, and blue tape) were mailed to participants' homes. Participants did not pay for access to the intervention and received a $50 gift card for study participation."

5-viii) Mode of delivery, features/functionalities/components of the intervention and comparator, and the theoretical framework

Describe mode of delivery, features/functionalities/components of the intervention and comparator, and the theoretical framework [6] used to design them (instructional strategy [1], behaviour change techniques, persuasive features, etc., see e.g., [7, 8] for terminology). This includes an in-depth description of the content (including where it is coming from and who developed it) [1]," whether [and how] it is tailored to individual circumstances and allows users to track their progress and receive feedback" [6]. This also includes a description of communication delivery channels and – if computer-mediated communication is a component – whether communication was synchronous or asynchronous [6]. It also includes information on presentation strategies [1], including page design principles, average amount of text on pages, presence of hyperlinks to other resources, etc. [1].

	1	2	3	4	5	
subitem not at all important	○	○	○	○	◉	essential

Clear selection

You're editing your response. Sharing this URL allows others to also edit your response. FILL OUT A NEW RESPONSE

Does your paper address subitem 5-viii? *

Copy and paste relevant sections from the manuscript (include quotes in quotation marks "like this" to indicate direct quotes from your manuscript), or elaborate on this item by providing additional information not in the ms, or briefly explain why the item is not applicable/relevant for your study

"The intervention combined synchronous supervised tele-exercise delivered via videoconferencing with asynchronous support through an mHealth platform that provided exercise videos and wearable-based monitoring. The intervention consisted of two phases: (1) an 8-week supervised phase (24 sessions; 3 sessions/week; ~50 minutes/session) delivered remotely; and (2) an 8-week independent home-based phase using the same platform. Exercise intensity was prescribed using an RIR-based RPE approach (target RPE 7–8) with standardized symptom-guided progression (modifying resistance, repetitions, range of motion, and rest based on fatigue, discomfort, or dizziness)."

5-ix) Describe use parameters

Describe use parameters (e.g., intended "doses" and optimal timing for use). Clarify what instructions or recommendations were given to the user, e.g., regarding timing, frequency, heaviness of use, if any, or was the intervention used ad libitum.

	1	2	3	4	5	
subitem not at all important	○	○	○	○	◉	essential

Clear selection

Does your paper address subitem 5-ix?

Copy and paste relevant sections from the manuscript (include quotes in quotation marks "like this" to indicate direct quotes from your manuscript), or elaborate on this item by providing additional information not in the ms, or briefly explain why the item is not applicable/relevant for your study

"Participants were prescribed three exercise sessions per week for 16 weeks, comprising 24 supervised sessions over weeks 1–8 followed by continued independent exercise sessions during weeks 9–16 using the same platform."

5-x) Clarify the level of human involvement

Clarify the level of human involvement (care providers or health professionals, also technical assistance) in the e-intervention or as co-intervention (detail number and expertise of professionals involved, if any, as well as "type of assistance offered, the timing and frequency of the support, how it is initiated, and the medium by which the assistance is delivered". It may be necessary to distinguish between the level of human involvement required for the trial, and the level of human involvement required for a routine application outside of a RCT setting (discuss under item 21 – generalizability).

	1	2	3	4	5	
subitem not at all important	○	○	○	○	◉	essential

Clear selection

Does your paper address subitem 5-x?

Copy and paste relevant sections from the manuscript (include quotes in quotation marks "like this" to indicate direct quotes from your manuscript), or elaborate on this item by providing additional information not in the ms, or briefly explain why the item is not applicable/relevant for your study

"Human involvement included remote supervision of all sessions during the first 8 weeks via videoconferencing. During the subsequent 8 weeks, participants exercised independently without direct monitoring by the exercise trainer, while adherence was tracked on the platform and participants remained engaged with the mHealth system."

5-xi) Report any prompts/reminders used

Report any prompts/reminders used: Clarify if there were prompts (letters, emails, phone calls, SMS) to use the application, what triggered them, frequency etc. It may be necessary to distinguish between the level of prompts/reminders required for the trial, and the level of prompts/reminders for a routine application outside of a RCT setting (discuss under item 21 – generalizability).

	1	2	3	4	5	
subitem not at all important	○	○	○	○	◉	essential

Clear selection

Does your paper address subitem 5-xi? *

Copy and paste relevant sections from the manuscript (include quotes in quotation marks "like this" to indicate direct quotes from your manuscript), or elaborate on this item by providing additional information not in the ms, or briefly explain why the item is not applicable/relevant for your study

"Adherence was tracked on the platform, and participants had the option to reschedule or make up a session if unable to attend as planned. Study staff maintained contact through the telehealth workflow to support attendance and completion of scheduled sessions."

You're editing your response. Sharing this URL allows others to also edit your response. FILL OUT A NEW RESPONSE

5-xii) Describe any co-interventions (incl. training/support)

Describe any co-interventions (incl. training/support): Clearly state any interventions that are provided in addition to the targeted eHealth intervention, as ehealth intervention may not be designed as stand-alone intervention. This includes training sessions and support [1]. It may be necessary to distinguish between the level of training required for the trial, and the level of training for a routine application outside of a RCT setting (discuss under item 21 – generalizability.

	1	2	3	4	5	
subitem not at all important	○	○	○	○	●	essential

Clear selection

Does your paper address subitem 5-xii? *

Copy and paste relevant sections from the manuscript (include quotes in quotation marks "like this" to indicate direct quotes from your manuscript), or elaborate on this item by providing additional information not in the ms, or briefly explain why the item is not applicable/relevant for your study

"Assessment equipment and exercise materials were mailed to participants, and all remote assessments were conducted under live videoconference supervision by trained research staff to ensure standardized administration. Participants in the control group were asked to maintain their current lifestyle for 17 weeks and were later offered the same mHealth exercise intervention as a courtesy after completing study procedures."

6a) Completely defined pre-specified primary and secondary outcome measures, including how and when they were assessed

Does your paper address CONSORT subitem 6a? *

Copy and paste relevant sections from the manuscript (include quotes in quotation marks "like this" to indicate direct quotes from your manuscript), or elaborate on this item by providing additional information not in the ms, or briefly explain why the item is not applicable/relevant for your study

"Remote assessments were conducted at baseline (week 0), midpoint (week 9), and follow-up (week 17). The primary outcome was change in the 5-component Fried frailty phenotype score (0–5). Secondary outcomes included SPPB components, chair-stand time, gait speed, and handgrip strength, all assessed remotely under videoconference supervision using mailed equipment."

6a-i) Online questionnaires: describe if they were validated for online use and apply CHERRIES items to describe how the questionnaires were designed/deployed

If outcomes were obtained through online questionnaires, describe if they were validated for online use and apply CHERRIES items to describe how the questionnaires were designed/deployed [9].

	1	2	3	4	5	
subitem not at all important	○	○	●	○	○	essential

Clear selection

Does your paper address subitem 6a-i?

Copy and paste relevant sections from manuscript text

"Not applicable. Outcomes were not collected via open online questionnaires; frailty and physical function assessments were conducted remotely under live videoconference supervision using mailed devices and standardized procedures."

6a-ii) Describe whether and how "use" (including intensity of use/dosage) was defined/measured/monitored

Describe whether and how "use" (including intensity of use/dosage) was defined/measured/monitored (logins, logfile analysis, etc.). Use/adoption metrics are important process outcomes that should be reported in any ehealth trial.

	1	2	3	4	5	
subitem not at all important	○	○	○	○	●	essential

Clear selection

Does your paper address subitem 6a-ii?

Copy and paste relevant sections from manuscript text

"Intervention use was defined as completion of prescribed exercise sessions and was measured as session adherence. Adherence during the supervised phase was calculated as completed videoconferencing sessions out of 24 prescribed sessions, and adherence during the independent phase was calculated as completed prescribed sessions tracked through the mHealth platform."

You're editing your response. Sharing this URL allows others to also edit your response. FILL OUT A NEW RESPONSE

6a-iii) Describe whether, how, and when qualitative feedback from participants was obtained

Describe whether, how, and when qualitative feedback from participants was obtained (e.g., through emails, feedback forms, interviews, focus groups).

	1	2	3	4	5	
subitem not at all important	○	○	◉	○	○	essential

Clear selection

Does your paper address subitem 6a-iii?

Copy and paste relevant sections from manuscript text

“Qualitative feedback was not collected using formal interviews or focus groups. Reasons for refusal during recruitment (eg, lack of interest, confidence in mHealth exercise settings, and time) were recorded as part of screening documentation.”

6b) Any changes to trial outcomes after the trial commenced, with reasons

Does your paper address CONSORT subitem 6b? *

Copy and paste relevant sections from the manuscript (include quotes in quotation marks "like this" to indicate direct quotes from your manuscript), or elaborate on this item by providing additional information not in the ms, or briefly explain why the item is not applicable/relevant for your study

“No changes to prespecified outcomes were made after trial commencement.”

7a) How sample size was determined

NPT: When applicable, details of whether and how the clustering by care provides or centers was addressed

7a-i) Describe whether and how expected attrition was taken into account when calculating the sample size

Describe whether and how expected attrition was taken into account when calculating the sample size.

	1	2	3	4	5	
subitem not at all important	○	○	○	○	◉	essential

Clear selection

Does your paper address subitem 7a-i?

Copy and paste relevant sections from manuscript title (include quotes in quotation marks "like this" to indicate direct quotes from your manuscript), or elaborate on this item by providing additional information not in the ms, or briefly explain why the item is not applicable/relevant for your study

"The original sample size calculation targeted a between-group difference in frailty phenotype score at week 9, assuming two-sided α=.05, 80% power, 20% attrition, and within-person correlation of 0.80 across repeated measures. A total of 60 participants (30 per group) were estimated to detect Cohen’s d=0.40. Due to recruitment challenges, the final sample was 32 participants (16 per group), providing 80% power to detect an effect size of approximately 0.50 under the same assumptions.”

7b) When applicable, explanation of any interim analyses and stopping guidelines

Does your paper address CONSORT subitem 7b? *

Copy and paste relevant sections from the manuscript (include quotes in quotation marks "like this" to indicate direct quotes from your manuscript), or elaborate on this item by providing additional information not in the ms, or briefly explain why the item is not applicable/relevant for your study

“Expected attrition of 20% was incorporated in the original sample size calculation.”

8a) Method used to generate the random allocation sequence

NPT: When applicable, how care providers were allocated to each trial group

Does your paper address CONSORT subitem 8a? *

Copy and paste relevant sections from the manuscript (include quotes in quotation marks "like this" to indicate direct quotes from your manuscript), or elaborate on this item by providing additional information not in the ms, or briefly explain why the item is not applicable/relevant for your study

“No interim analyses or stopping guidelines were planned.”

8b) Type of randomisation; details of any restriction (such as blocking and block size)

You're editing your response. Sharing this URL allows others to also edit your response.

FILL OUT A NEW RESPONSE

Does your paper address CONSORT subitem 8b? *

Copy and paste relevant sections from the manuscript (include quotes in quotation marks "like this" to indicate direct quotes from your manuscript), or elaborate on this item by providing additional information not in the ms, or briefly explain why the item is not applicable/relevant for your study

“Participants were randomly assigned in a 1:1 ratio using a computer-generated random allocation sequence prepared by an independent statistician not involved in study conduct.”

9) Mechanism used to implement the random allocation sequence (such as sequentially numbered containers), describing any steps taken to conceal the sequence until interventions were assigned

Does your paper address CONSORT subitem 9? *

Copy and paste relevant sections from the manuscript (include quotes in quotation marks "like this" to indicate direct quotes from your manuscript), or elaborate on this item by providing additional information not in the ms, or briefly explain why the item is not applicable/relevant for your study

“Randomization was conducted with a 1:1 allocation ratio; no additional restrictions such as blocking or stratification were reported.”

10) Who generated the random allocation sequence, who enrolled participants, and who assigned participants to interventions

Does your paper address CONSORT subitem 10? *

Copy and paste relevant sections from the manuscript (include quotes in quotation marks "like this" to indicate direct quotes from your manuscript), or elaborate on this item by providing additional information not in the ms, or briefly explain why the item is not applicable/relevant for your study

“Allocation concealment was maintained: participants were enrolled by the study coordinator, while group assignments were implemented by a separate investigator who was unaware of the allocation sequence.”

11a) If done, who was blinded after assignment to interventions (for example, participants, care providers, those assessing outcomes) and how

NPT: Whether or not administering co-interventions were blinded to group assignment

11a-i) Specify who was blinded, and who wasn't

Specify who was blinded, and who wasn't. Usually, in web-based trials it is not possible to blind the participants [1, 3] (this should be clearly acknowledged), but it may be possible to blind outcome assessors, those doing data analysis or those administering co-interventions (if any).

	1	2	3	4	5	
subitem not at all important	○	○	○	◉	○	essential

Clear selection

Does your paper address subitem 11a-i? *

Copy and paste relevant sections from the manuscript (include quotes in quotation marks "like this" to indicate direct quotes from your manuscript), or elaborate on this item by providing additional information not in the ms, or briefly explain why the item is not applicable/relevant for your study

“Due to the nature of the exercise intervention, participants and intervention staff were not blinded. Remote assessments were conducted under standardized videoconference procedures by trained research staff; blinding of assessors was not specified and is not feasible to fully ensure in telehealth-delivered exercise trials.”

11a-ii) Discuss e.g., whether participants knew which intervention was the “intervention of interest” and which one was the “comparator”

Informed consent procedures (4a-ii) can create biases and certain expectations - discuss e.g., whether participants knew which intervention was the “intervention of interest” and which one was the “comparator”.

	1	2	3	4	5	
subitem not at all important	○	○	○	◉	○	essential

Clear selection

Does your paper address subitem 11a-ii?

Copy and paste relevant sections from the manuscript (include quotes in quotation marks "like this" to indicate direct quotes from your manuscript), or elaborate on this item by providing additional information not in the ms, or briefly explain why the item is not applicable/relevant for your study

“Participants were informed during consent that they would be randomized to an exercise intervention or usual care. The control group was offered the intervention after completing follow-up assessments, which may have influenced expectations; this limitation was acknowledged given the lack of an attention-matched control condition.”

You're editing your response. Sharing this URL allows others to also edit your response.

FILL OUT A NEW RESPONSE

11b) If relevant, description of the similarity of interventions
(this item is usually not relevant for ehealth trials as it refers to similarity of a placebo or sham intervention to a active medication/intervention)

Does your paper address CONSORT subitem 11b? *
Copy and paste relevant sections from the manuscript (include quotes in quotation marks "like this" to indicate direct quotes from your manuscript), or elaborate on this item by providing additional information not in the ms, or briefly explain why the item is not applicable/relevant for your study

"Not applicable. The comparator was usual care without a structured exercise rehabilitation program."

12a) Statistical methods used to compare groups for primary and secondary outcomes
NPT: When applicable, details of whether and how the clustering by care providers or centers was addressed

Does your paper address CONSORT subitem 12a? *
Copy and paste relevant sections from the manuscript (include quotes in quotation marks "like this" to indicate direct quotes from your manuscript), or elaborate on this item by providing additional information not in the ms, or briefly explain why the item is not applicable/relevant for your study

"Intention-to-treat analyses were performed using generalized estimating equations (GEE) with two indicators of time, assuming a compound symmetry within-person correlation matrix. Treatment-by-time interaction was included and tested using a 2-df χ^2 test. Models were adjusted a priori for body mass index, age, and race (non-Hispanic White vs other). Between-group differences at week 17 with 95% confidence intervals were computed and compared with week 9 differences."

12a-i) Imputation techniques to deal with attrition / missing values
Imputation techniques to deal with attrition / missing values: Not all participants will use the intervention/comparator as intended and attrition is typically high in ehealth trials. Specify how participants who did not use the application or dropped out from the trial were treated in the statistical analysis (a complete case analysis is strongly discouraged, and simple imputation techniques such as LOCF may also be problematic [4]).

	1	2	3	4	5	
subitem not at all important	○	○	○	○	◉	essential

Clear selection

Does your paper address subitem 12a-i? *
Copy and paste relevant sections from the manuscript (include quotes in quotation marks "like this" to indicate direct quotes from your manuscript), or elaborate on this item by providing additional information not in the ms, or briefly explain why the item is not applicable/relevant for your study

"GEE analyses were conducted under an intention-to-treat framework using all randomized participants. No single imputation method (eg, LOCF) was used; inference relied on the repeated-measures GEE approach with available data and prespecified covariate adjustment."

12b) Methods for additional analyses, such as subgroup analyses and adjusted analyses

Does your paper address CONSORT subitem 12b? *
Copy and paste relevant sections from the manuscript (include quotes in quotation marks "like this" to indicate direct quotes from your manuscript), or elaborate on this item by providing additional information not in the ms, or briefly explain why the item is not applicable/relevant for your study

"Adjusted between-group differences at week 9 and week 17 were estimated from the same GEE models and reported with 95% confidence intervals."

X26) REB/IRB Approval and Ethical Considerations [recommended as subheading under "Methods"] (not a CONSORT item)

X26-i) Comment on ethics committee approval

	1	2	3	4	5	
subitem not at all important	○	○	○	○	◉	essential

Clear selection

Does your paper address subitem X26-i?
Copy and paste relevant sections from the manuscript (include quotes in quotation marks "like this" to indicate direct quotes from your manuscript), or elaborate on this item by providing additional information not in the ms, or briefly explain why the item is not applicable/relevant for your study

"The study protocol was approved by the City of Hope Institutional Review Board (IRB# 21406)."

You're editing your response. Sharing this URL allows others to also edit your response. FILL OUT A NEW RESPONSE

x26-ii) Outline informed consent procedures

Outline informed consent procedures e.g., if consent was obtained offline or online (how? Checkbox, etc.?), and what information was provided (see 4a-ii). See [6] for some items to be included in informed consent documents.

	1	2	3	4	5	
subitem not at all important	○	○	○	○	◉	essential

Clear selection

Does your paper address subitem X26-ii?

Copy and paste relevant sections from the manuscript (include quotes in quotation marks "like this" to indicate direct quotes from your manuscript), or elaborate on this item by providing additional information not in the ms, or briefly explain why the item is not applicable/relevant for your study

"All participants provided written informed consent electronically using DocuSign™ prior to participation and could withdraw at any time without affecting their clinical care."

X26-iii) Safety and security procedures

Safety and security procedures, incl. privacy considerations, and any steps taken to reduce the likelihood or detection of harm (e.g., education and training, availability of a hotline)

	1	2	3	4	5	
subitem not at all important	○	○	○	○	◉	essential

Clear selection

Does your paper address subitem X26-iii?

Copy and paste relevant sections from the manuscript (include quotes in quotation marks "like this" to indicate direct quotes from your manuscript), or elaborate on this item by providing additional information not in the ms, or briefly explain why the item is not applicable/relevant for your study

"Safety procedures included screening for contraindications to exercise, physician clearance via the primary hematologist when indicated, live videoconference supervision by trained staff for remote assessments, and symptom-guided modification of exercise intensity and progression. Study data were de-identified and stored on secure institutional servers accessible only to authorized study personnel."

RESULTS

13a) For each group, the numbers of participants who were randomly assigned, received intended treatment, and were analysed for the primary outcome

NPT: The number of care providers or centers performing the intervention in each group and the number of patients treated by each care provider in each center

Does your paper address CONSORT subitem 13a? *

Copy and paste relevant sections from the manuscript (include quotes in quotation marks "like this" to indicate direct quotes from your manuscript), or elaborate on this item by providing additional information not in the ms, or briefly explain why the item is not applicable/relevant for your study

"Of 33 enrolled pre-frail or frail patients, one withdrew prior to randomization, resulting in 32 participants randomized to the exercise (n=16) or control (n=16) group. Intention-to-treat analyses were performed using all randomized participants."

13b) For each group, losses and exclusions after randomisation, together with reasons

Does your paper address CONSORT subitem 13b? (NOTE: Preferably, this is shown in a CONSORT flow diagram) *

Copy and paste relevant sections from the manuscript (include quotes in quotation marks "like this" to indicate direct quotes from your manuscript), or elaborate on this item by providing additional information not in the ms, or briefly explain why the item is not applicable/relevant for your study

"At week 9, 2/32 (6%) participants were lost to follow-up, and an additional 2/30 (7%) were lost at week 17 (one from each group at both timepoints). Reasons for refusal and ineligibility during recruitment are summarized in the participant flow (Figure 2)."

13b-i) Attrition diagram

Strongly recommended: An attrition diagram (e.g., proportion of participants still logging in or using the intervention/comparator in each group plotted over time, similar to a survival curve) or other figures or tables demonstrating usage/dose/engagement.

	1	2	3	4	5	
subitem not at all important	○	○	○	○	◉	essential

Clear selection

You're editing your response. Sharing this URL allows others to also edit your response. FILL OUT A NEW RESPONSE

Does your paper address subitem 13b-i?

Copy and paste relevant sections from the manuscript or cite the figure number if applicable (include quotes in quotation marks "like this" to indicate direct quotes from your manuscript), or elaborate on this item by providing additional information not in the ms, or briefly explain why the item is not applicable/relevant for your study

"Engagement was quantified using session adherence during the supervised phase (326/384 sessions, 85%) and completion of prescribed sessions during the independent phase (298/384 sessions, 78%). These metrics represent intensity of use over time."

14a) Dates defining the periods of recruitment and follow-up

Does your paper address CONSORT subitem 14a? *

Copy and paste relevant sections from the manuscript (include quotes in quotation marks "like this" to indicate direct quotes from your manuscript), or elaborate on this item by providing additional information not in the ms, or briefly explain why the item is not applicable/relevant for your study

"The trial was registered on ClinicalTrials.gov (NCT05142371) on October 29, 2021; recruitment and follow-up occurred within the study period described in the registered protocol and trial conduct at City of Hope."

14a-i) Indicate if critical "secular events" fell into the study period

Indicate if critical "secular events" fell into the study period, e.g., significant changes in Internet resources available or "changes in computer hardware or Internet delivery resources"

	1	2	3	4	5	
subitem not at all important	○	○	○	○	◉	essential

Clear selection

Does your paper address subitem 14a-i?

Copy and paste relevant sections from the manuscript (include quotes in quotation marks "like this" to indicate direct quotes from your manuscript), or elaborate on this item by providing additional information not in the ms, or briefly explain why the item is not applicable/relevant for your study

"No critical secular events occurred during the study period that affected access to internet resources, videoconferencing capability, wearable device availability, or delivery of the mHealth platform. There were no changes in computer hardware requirements, internet delivery resources, or platform functionality that required modification of study procedures."

14b) Why the trial ended or was stopped (early)

Does your paper address CONSORT subitem 14b? *

Copy and paste relevant sections from the manuscript (include quotes in quotation marks "like this" to indicate direct quotes from your manuscript), or elaborate on this item by providing additional information not in the ms, or briefly explain why the item is not applicable/relevant for your study

"The trial ended as planned after completion of the 16-week intervention and scheduled remote assessments through week 17."

15) A table showing baseline demographic and clinical characteristics for each group

NPT: When applicable, a description of care providers (case volume, qualification, expertise, etc.) and centers (volume) in each group

Does your paper address CONSORT subitem 15? *

Copy and paste relevant sections from the manuscript (include quotes in quotation marks "like this" to indicate direct quotes from your manuscript), or elaborate on this item by providing additional information not in the ms, or briefly explain why the item is not applicable/relevant for your study

"Baseline demographic and clinical characteristics are presented in Table 1, including age, sex, body mass index, race/ethnicity, comorbidities, baseline physical activity, and baseline frailty components."

15-i) Report demographics associated with digital divide issues

In ehealth trials it is particularly important to report demographics associated with digital divide issues, such as age, education, gender, social-economic status, computer/Internet/ehealth literacy of the participants, if known.

	1	2	3	4	5	
subitem not at all important	○	○	○	○	◉	essential

Clear selection

You're editing your response. Sharing this URL allows others to also edit your response.

FILL OUT A NEW RESPONSE

Does your paper address subitem 15-i? *

Copy and paste relevant sections from the manuscript (include quotes in quotation marks "like this" to indicate direct quotes from your manuscript), or elaborate on this item by providing additional information not in the ms, or briefly explain why the item is not applicable/relevant for your study

"Demographic characteristics relevant to digital access, including age, sex, race/ethnicity, and baseline physical activity, were reported in Table 1. Eligibility required English-speaking participation, which may limit generalizability to individuals with lower digital literacy or non-English speakers."

16) For each group, number of participants (denominator) included in each analysis and whether the analysis was by original assigned groups

16-i) Report multiple "denominators" and provide definitions

Report multiple "denominators" and provide definitions: Report N's (and effect sizes) "across a range of study participation [and use] thresholds" [1], e.g., N exposed, N consented, N used more than x times, N used more than y weeks, N participants "used" the intervention/comparator at specific pre-defined time points of interest (in absolute and relative numbers per group). Always clearly define "use" of the intervention.

	1	2	3	4	5	
subitem not at all important	○	○	○	○	◉	essential

Clear selection

Does your paper address subitem 16-i? *

Copy and paste relevant sections from the manuscript (include quotes in quotation marks "like this" to indicate direct quotes from your manuscript), or elaborate on this item by providing additional information not in the ms, or briefly explain why the item is not applicable/relevant for your study

"Denominators were reported for potentially eligible patients identified (n=212), successfully contacted (n=129), refusals (n=46), ineligible patients (n=45), enrolled (n=33), randomized (n=32), and those completing follow-up assessments at week 9 and week 17. Intervention 'use' was defined as completion of prescribed exercise sessions and quantified using adherence metrics for each intervention phase."

16-ii) Primary analysis should be intent-to-treat

Primary analysis should be intent-to-treat, secondary analyses could include comparing only "users", with the appropriate caveats that this is no longer a randomized sample (see 18-i).

	1	2	3	4	5	
subitem not at all important	○	○	○	○	◉	essential

Clear selection

Does your paper address subitem 16-ii?

Copy and paste relevant sections from the manuscript (include quotes in quotation marks "like this" to indicate direct quotes from your manuscript), or elaborate on this item by providing additional information not in the ms, or briefly explain why the item is not applicable/relevant for your study

"Primary analyses were conducted using intention-to-treat methods, including all randomized participants according to original group assignment."

17a) For each primary and secondary outcome, results for each group, and the estimated effect size and its precision (such as 95% confidence interval)

Does your paper address CONSORT subitem 17a? *

Copy and paste relevant sections from the manuscript (include quotes in quotation marks "like this" to indicate direct quotes from your manuscript), or elaborate on this item by providing additional information not in the ms, or briefly explain why the item is not applicable/relevant for your study

"Process outcomes included adherence to supervised sessions (326/384 sessions, 85%) and adherence during the independent phase (298/384 sessions, 78%), with operational definitions based on completion of prescribed sessions tracked through the mHealth platform."

17a-i) Presentation of process outcomes such as metrics of use and intensity of use

In addition to primary/secondary (clinical) outcomes, the presentation of process outcomes such as metrics of use and intensity of use (dose, exposure) and their operational definitions is critical. This does not only refer to metrics of attrition (13-b) (often a binary variable), but also to more continuous exposure metrics such as "average session length". These must be accompanied by a technical description how a metric like a "session" is defined (e.g., timeout after idle time) [1] (report under item 6a).

	1	2	3	4	5	
subitem not at all important	○	○	○	○	◉	essential

Clear selection

You're editing your response. Sharing this URL allows others to also edit your response.

FILL OUT A NEW RESPONSE

Does your paper address subitem 17a-i?

Copy and paste relevant sections from the manuscript (include quotes in quotation marks "like this" to indicate direct quotes from your manuscript), or elaborate on this item by providing additional information not in the ms, or briefly explain why the item is not applicable/relevant for your study

"Process outcomes included adherence to supervised sessions (326/384 sessions, 85%) and adherence during the independent phase (298/384 sessions, 78%), with operational definitions based on completion of prescribed sessions tracked through the mHealth platform."

17b) For binary outcomes, presentation of both absolute and relative effect sizes is recommended

Does your paper address CONSORT subitem 17b? *

Copy and paste relevant sections from the manuscript (include quotes in quotation marks "like this" to indicate direct quotes from your manuscript), or elaborate on this item by providing additional information not in the ms, or briefly explain why the item is not applicable/relevant for your study

"This item is not applicable to our study. The primary and secondary outcomes were analyzed and reported as continuous variables (frailty phenotype score, chair-stand time, gait speed, handgrip strength, and SPPB score). As presented in the manuscript, between-group differences were reported as adjusted mean differences with 95% confidence intervals derived from generalized estimating equation models (Table 2). No binary outcomes were analyzed; therefore, reporting of absolute and relative effect sizes for binary outcomes does not apply to this study."

18) Results of any other analyses performed, including subgroup analyses and adjusted analyses, distinguishing pre-specified from exploratory

Does your paper address CONSORT subitem 18? *

Copy and paste relevant sections from the manuscript (include quotes in quotation marks "like this" to indicate direct quotes from your manuscript), or elaborate on this item by providing additional information not in the ms, or briefly explain why the item is not applicable/relevant for your study

Adjusted analyses were pre-specified in the statistical analysis plan. As described in the Methods, 'Changes in frailty index from baseline to week 9 and week 17 were evaluated using the Generalized Estimation Equation (GEE) methods… Body mass index, age, and race (non-Hispanic white vs other) were included as a priori covariates for adjustment.' No subgroup analyses were pre-specified or conducted. All analyses followed the intention-to-treat principle based on original group assignment. Therefore, results presented reflect pre-specified adjusted analyses rather than exploratory subgroup analyses."

18-i) Subgroup analysis of comparing only users

A subgroup analysis of comparing only users is not uncommon in ehealth trials, but if done, it must be stressed that this is a self-selected sample and no longer an unbiased sample from a randomized trial (see 16-iii).

	1	2	3	4	5	
subitem not at all important	◉	○	○	○	○	essential

Clear selection

Does your paper address subitem 18-i?

Copy and paste relevant sections from the manuscript (include quotes in quotation marks "like this" to indicate direct quotes from your manuscript), or elaborate on this item by providing additional information not in the ms, or briefly explain why the item is not applicable/relevant for your study

"This item is not applicable to our study. No subgroup analyses restricted to 'users only' were conducted. All analyses followed the intention-to-treat principle based on original group assignment. As stated in the manuscript: "Assuming a two-sided Type I error=0.05, intention-to-treat analyses were performed using all randomized participants." Additionally, "GEE analyses were conducted under an intention-to-treat framework using all randomized participants." Therefore, no per-protocol or adherence-restricted analyses were used to estimate intervention effects."

19) All important harms or unintended effects in each group
(for specific guidance see CONSORT for harms)

Does your paper address CONSORT subitem 19? *

Copy and paste relevant sections from the manuscript (include quotes in quotation marks "like this" to indicate direct quotes from your manuscript), or elaborate on this item by providing additional information not in the ms, or briefly explain why the item is not applicable/relevant for your study

"No serious adverse events or unintended effects were reported during the intervention period."

You're editing your response. Sharing this URL allows others to also edit your response. FILL OUT A NEW RESPONSE

19-i) Include privacy breaches, technical problems

Include privacy breaches, technical problems. This does not only include physical "harm" to participants, but also incidents such as perceived or real privacy breaches [1], technical problems, and other unexpected/unintended incidents. "Unintended effects" also includes unintended positive effects [2].

	1	2	3	4	5	
subitem not at all important	○	○	○	◉	○	essential

Clear selection

Does your paper address subitem 19-i?

Copy and paste relevant sections from the manuscript (include quotes in quotation marks "like this" to indicate direct quotes from your manuscript), or elaborate on this item by providing additional information not in the ms, or briefly explain why the item is not applicable/relevant for your study

"No privacy breaches, technical problems, or platform-related unintended effects were reported."

19-ii) Include qualitative feedback from participants or observations from staff/researchers

Include qualitative feedback from participants or observations from staff/researchers, if available, on strengths and shortcomings of the application, especially if they point to unintended/unexpected effects or uses. This includes (if available) reasons for why people did or did not use the application as intended by the developers.

	1	2	3	4	5	
subitem not at all important	○	○	◉	○	○	essential

Clear selection

Does your paper address subitem 19-ii?

Copy and paste relevant sections from the manuscript (include quotes in quotation marks "like this" to indicate direct quotes from your manuscript), or elaborate on this item by providing additional information not in the ms, or briefly explain why the item is not applicable/relevant for your study

"Formal qualitative feedback was not collected; however, recruitment documentation captured common reasons for refusal, including lack of interest, confidence in mHealth exercise settings, and time."

DISCUSSION

22) Interpretation consistent with results, balancing benefits and harms, and considering other relevant evidence

NPT: In addition, take into account the choice of the comparator, lack of or partial blinding, and unequal expertise of care providers or centers in each group

22-i) Restate study questions and summarize the answers suggested by the data, starting with primary outcomes and process outcomes (use)

Restate study questions and summarize the answers suggested by the data, starting with primary outcomes and process outcomes (use).

	1	2	3	4	5	
subitem not at all important	○	○	○	◉	○	essential

Clear selection

Does your paper address subitem 22-i? *

Copy and paste relevant sections from the manuscript (include quotes in quotation marks "like this" to indicate direct quotes from your manuscript), or elaborate on this item by providing additional information not in the ms, or briefly explain why the item is not applicable/relevant for your study

"This trial evaluated whether a 16-week mHealth-supported exercise rehabilitation program could improve frailty phenotype and physical function in pre-frail or frail multiple myeloma survivors within 180 days after autologous HCT. The primary outcome showed a significant between-group reduction in frailty phenotype score favoring the exercise group at both week 9 and week 17. Process outcomes demonstrated high engagement, with 85% adherence during the supervised phase and 78% adherence during the independent phase. Secondary outcomes showed significant improvements in chair-stand performance, with favorable trends in other physical function measures. These findings indicate that a remotely delivered, progressively prescribed exercise program was feasible, safe, and effective in reversing frailty and improving functional mobility in this high-risk population."

22-ii) Highlight unanswered new questions, suggest future research

Highlight unanswered new questions, suggest future research.

	1	2	3	4	5	
subitem not at all important	○	○	○	◉	○	essential

Clear selection

You're editing your response. Sharing this URL allows others to also edit your response. FILL OUT A NEW RESPONSE

Does your paper address subitem 22-ii?

Copy and paste relevant sections from the manuscript (include quotes in quotation marks "like this" to indicate direct quotes from your manuscript), or elaborate on this item by providing additional information not in the ms, or briefly explain why the item is not applicable/relevant for your study

"Future research should evaluate the scalability of this mHealth-supported rehabilitation model across multiple centers and diverse oncology populations, incorporate attention-matched control conditions to better isolate exercise-specific effects, and examine the long-term sustainability of frailty reversal beyond the supervised period. Additional studies are needed to determine optimal strategies for reducing human resource demands while maintaining adherence, and to explore whether similar benefits can be achieved in individuals with lower digital literacy or in non-English-speaking populations."

20) Trial limitations, addressing sources of potential bias, imprecision, and, if relevant, multiplicity of analyses

20-i) Typical limitations in ehealth trials

Typical limitations in ehealth trials: Participants in ehealth trials are rarely blinded. Ehealth trials often look at a multiplicity of outcomes, increasing risk for a Type I error. Discuss biases due to non-use of the intervention/usability issues, biases through informed consent procedures, unexpected events.

	1	2	3	4	5	
subitem not at all important	○	○	○	○	◉	essential

Clear selection

Does your paper address subitem 20-i? *

Copy and paste relevant sections from the manuscript (include quotes in quotation marks "like this" to indicate direct quotes from your manuscript), or elaborate on this item by providing additional information not in the ms, or briefly explain why the item is not applicable/relevant for your study

"This study has several limitations. First, the modest sample size and single-center design limit statistical precision and generalizability. Second, the control group did not receive an attention-matched intervention; therefore, non-specific effects related to increased contact, accountability, or health-focused reminders cannot be excluded as contributors to the observed improvements. Third, eligibility required English proficiency and the ability to participate in telehealth-based procedures, which may limit applicability to individuals with lower digital literacy or non-English speakers. Fourth, although objective remote assessments were incorporated using mailed equipment and videoconference supervision, some frailty components relied on self-reported measures, which may introduce reporting bias. Finally, remote assessments conducted in home environments may have introduced variability in testing conditions compared with standardized in-person assessments."

21) Generalisability (external validity, applicability) of the trial findings

NPT: External validity of the trial findings according to the intervention, comparators, patients, and care providers or centers involved in the trial

21-i) Generalizability to other populations

Generalizability to other populations: In particular, discuss generalizability to a general Internet population, outside of a RCT setting, and general patient population, including applicability of the study results for other organizations

	1	2	3	4	5	
subitem not at all important	○	○	○	○	◉	essential

Clear selection

Does your paper address subitem 21-i?

Copy and paste relevant sections from the manuscript (include quotes in quotation marks "like this" to indicate direct quotes from your manuscript), or elaborate on this item by providing additional information not in the ms, or briefly explain why the item is not applicable/relevant for your study

"Findings may generalize to similar post-transplant oncology populations with limited access to center-based rehabilitation where telehealth delivery and mailed equipment-based remote assessment are feasible; however, applicability may be reduced for non-English speakers and individuals with lower digital literacy."

21-ii) Discuss if there were elements in the RCT that would be different in a routine application setting

Discuss if there were elements in the RCT that would be different in a routine application setting (e.g., prompts/reminders, more human involvement, training sessions or other co-interventions) and what impact the omission of these elements could have on use, adoption, or outcomes if the intervention is applied outside of a RCT setting.

	1	2	3	4	5	
subitem not at all important	○	○	○	○	◉	essential

Clear selection

You're editing your response. Sharing this URL allows others to also edit your response.

FILL OUT A NEW RESPONSE

Does your paper address subitem 21-ii?

Copy and paste relevant sections from the manuscript (include quotes in quotation marks "like this" to indicate direct quotes from your manuscript), or elaborate on this item by providing additional information not in the ms, or briefly explain why the item is not applicable/relevant for your study

"Implementation outside an RCT may differ in intensity of human support, frequency of videoconference supervision, and provision of mailed equipment; reductions in these elements could affect adherence and outcomes."

OTHER INFORMATION

23) Registration number and name of trial registry

Does your paper address CONSORT subitem 23? *

Copy and paste relevant sections from the manuscript (include quotes in quotation marks "like this" to indicate direct quotes from your manuscript), or elaborate on this item by providing additional information not in the ms, or briefly explain why the item is not applicable/relevant for your study

"ClinicalTrials.gov NCT05142371."

24) Where the full trial protocol can be accessed, if available

Does your paper address CONSORT subitem 24? *

Cite a Multimedia Appendix, other reference, or copy and paste relevant sections from the manuscript (include quotes in quotation marks "like this" to indicate direct quotes from your manuscript), or elaborate on this item by providing additional information not in the ms, or briefly explain why the item is not applicable/relevant for your study

"The trial protocol has been published (Telehealth exercise to Improve Physical function and frailty in patients with multiple myeloma treated with autologous hematopoietic stem cell transplantation [TIPS]: protocol of a randomized controlled trial)."

25) Sources of funding and other support (such as supply of drugs), role of funders

Does your paper address CONSORT subitem 25? *

Copy and paste relevant sections from the manuscript (include quotes in quotation marks "like this" to indicate direct quotes from your manuscript), or elaborate on this item by providing additional information not in the ms, or briefly explain why the item is not applicable/relevant for your study

"This study was supported by the 2021 Steven Gordon and Briskin Family Innovation Grant. The funding source had no role in the design of the study; data collection, analysis, or interpretation; manuscript preparation; or the decision to submit the manuscript for publication."

X27) Conflicts of Interest (not a CONSORT item)

X27-i) State the relation of the study team towards the system being evaluated

In addition to the usual declaration of interests (financial or otherwise), also state the relation of the study team towards the system being evaluated, i.e., state if the authors/evaluators are distinct from or identical with the developers/sponsors of the intervention.

	1	2	3	4	5	
subitem not at all important	○	○	○	○	◉	essential

Clear selection

Does your paper address subitem X27-i?

Copy and paste relevant sections from the manuscript (include quotes in quotation marks "like this" to indicate direct quotes from your manuscript), or elaborate on this item by providing additional information not in the ms, or briefly explain why the item is not applicable/relevant for your study

"The intervention used a commercially available platform (Moterum Technologies) and the study team was independent of the commercial platform ownership. The investigators developed the exercise prescription and remote assessment procedures but declare no conflicts of interest related to the evaluated system."

About the CONSORT EHEALTH checklist

As a result of using this checklist, did you make changes in your manuscript? *

- ○ yes, major changes
- ◉ yes, minor changes
- ○ no

You're editing your response. Sharing this URL allows others to also edit your response. FILL OUT A NEW RESPONSE

What were the most important changes you made as a result of using this checklist?

Title and abstract are the most

How much time did you spend on going through the checklist INCLUDING making changes in your manuscript *

30 plus hours of going through the checklist and making changes

As a result of using this checklist, do you think your manuscript has improved? *

- (x) yes
- ( ) no
- ( ) Other:

Would you like to become involved in the CONSORT EHEALTH group?

This would involve for example becoming involved in participating in a workshop and writing an "Explanation and Elaboration" document

- ( ) yes
- (x) no
- ( ) Other:

Clear selection

Any other comments or questions on CONSORT EHEALTH

Your answer

STOP - Save this form as PDF before you click submit

To generate a record that you filled in this form, we recommend to generate a PDF of this page (on a Mac, simply select "print" and then select "print as PDF") before you submit it.

When you submit your (revised) paper to JMIR, please upload the PDF as supplementary file.

Don't worry if some text in the textboxes is cut off, as we still have the complete information in our database. Thank you!

Final step: Click submit !

Click submit so we have your answers in our database!

Submit

Never submit passwords through Google Forms.

This content is neither created nor endorsed by Google. - Contact form owner - Terms of Service - Privacy Policy

Does this form look suspicious? Report

Google Forms

You're editing your response. Sharing this URL allows others to also edit your response.

FILL OUT A NEW RESPONSE

You're editing your response. Sharing this URL allows others to also edit your response.
FILL OUT A NEW RESPONSE
